# Modeling of the axon plasma membrane structure and its effects on protein diffusion

**DOI:** 10.1371/journal.pcbi.1007003

**Published:** 2019-05-02

**Authors:** Yihao Zhang, Anastasios V. Tzingounis, George Lykotrafitis

**Affiliations:** 1 Department of Mechanical Engineering, University of Connecticut, Storrs, CT, United States of America; 2 Department of Physiology and Neurobiology, University of Connecticut, Storrs, CT, United States of America; 3 Department of Biomedical Engineering, University of Connecticut, Storrs, CT, United States of America; Icahn School of Medicine at Mount Sinai, UNITED STATES

## Abstract

The axon plasma membrane consists of the membrane skeleton, which comprises ring-like actin filaments connected to each other by spectrin tetramers, and the lipid bilayer, which is tethered to the skeleton via, at least, ankyrin. Currently it is unknown whether this unique axon plasma membrane skeleton (APMS) sets the diffusion rules of lipids and proteins in the axon. To answer this question, we developed a coarse-grain molecular dynamics model for the axon that includes the APMS, the phospholipid bilayer, transmembrane proteins (TMPs), and integral monotopic proteins (IMPs) in both the inner and outer lipid layers. We first showed that actin rings limit the longitudinal diffusion of TMPs and the IMPs of the inner leaflet but not of the IMPs of the outer leaflet. To reconcile the experimental observations, which show restricted diffusion of IMPs of the outer leaflet, with our simulations, we conjectured the existence of actin-anchored proteins that form a fence which restricts the longitudinal diffusion of IMPs of the outer leaflet. We also showed that spectrin filaments could modify transverse diffusion of TMPs and IMPs of the inner leaflet, depending on the strength of the association between lipids and spectrin. For instance, in areas where spectrin binds to the lipid bilayer, spectrin filaments would restrict diffusion of proteins within the skeleton corrals. In contrast, in areas where spectrin and lipids are not associated, spectrin modifies the diffusion of TMPs and IMPs of the inner leaflet from normal to confined-hop diffusion. Overall, we showed that diffusion of axon plasma membrane proteins is deeply anisotropic, as longitudinal diffusion is of different type than transverse diffusion. Finally, we investigated how accumulation of TMPs affects diffusion of TMPs and IMPs of both the inner and outer leaflets by changing the density of TMPs. We showed that the APMS structure acts as a fence that restricts the diffusion of TMPs and IMPs of the inner leaflet within the membrane skeleton corrals. Our findings provide insight into how the axon skeleton acts as diffusion barrier and maintains neuronal polarity.

## Author summary

The axon plasma membrane skeleton consists of repeated periodic actin ring-like structures along its length connected via spectrin tetramers and anchored to the lipid bilayer at least via ankyrin. However, it is currently unclear whether this structure controls diffusion of lipids and proteins in the axon. Here, we developed a coarse-grain molecular dynamics computational model for the axon plasma membrane that comprises minimal representations for the APMS and the lipid bilayer. In a departure from current models, we found that actin rings limit diffusion of proteins only in the inner membrane leaflet. Then, we showed that actin anchored proteins likely act as “fences” confining diffusion of proteins in the outer leaflet. Our simulations, unexpectedly, also revealed that spectrin filaments could impede transverse diffusion in the inner leaflet of the axon and in some conditions modify diffusion from normal to abnormal. We predicted that diffusion of axon plasma membrane proteins is anisotropic as longitudinal diffusion is of different type than transverse (azimuthal) diffusion. We conclude that the periodic structure of the axon plays a critical role in controlling diffusion of proteins and lipids in the axon plasma membrane.

## Introduction

**Fig 1:**
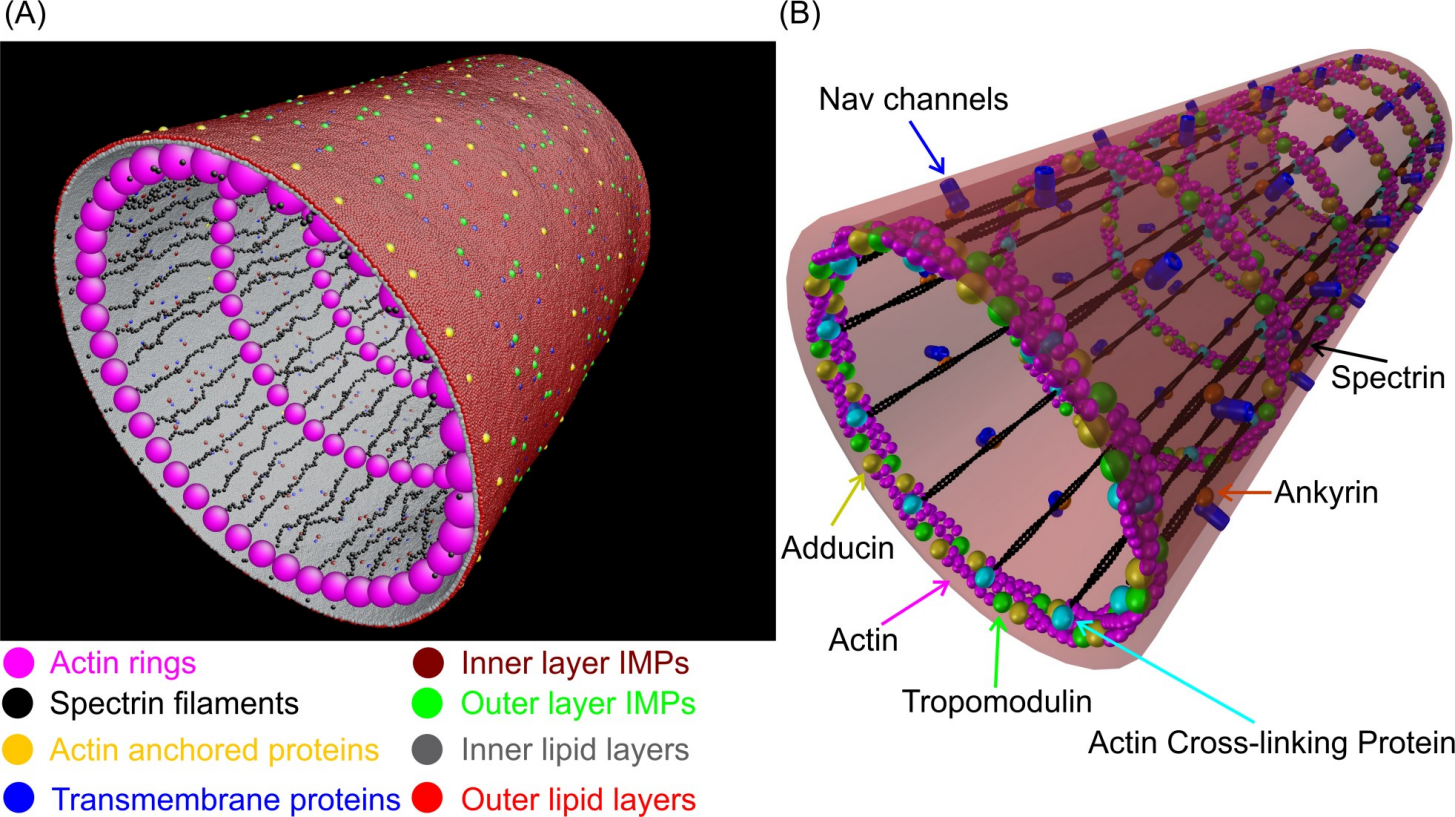
Computational and schematic model of the APM. (A) Computational model of the APM showing different structural components. (B) Schematic illustration of the APM based on super-resolution microscopy results of the periodic APMS structure. Ankyrin-associated Na_v_ channels anchor the phospholipid bilayer to the membrane skeleton.

A neuron is an electrically excitable and highly polarized cell that primarily functions to receive, integrate, and transmit information. A neuron is comprised of three main compartments: a soma, dendrites, and an axon [1]. A key aspect of neuronal function is the integration of arriving synaptic potentials, and generation and propagation of action potentials down a single axon [2, 3]. Multiple studies have shown that axons are cylindrical structures consisting of the axon plasma membrane (APM) and cytoplasm [4]. The APM is composed of two main substructures: the phospholipid bilayer, which contains ion channels and other membrane proteins, and the membrane skeleton that tethers to the phospholipid bilayer (Fig 1). The membrane cytoskeleton is also connected to axon microtubules via ankyrin proteins [5]. Recent research revealed that the axon plasma membrane skeleton (APMS) has a unique long-range periodic structure that is comprised of a series of actin rings distributed along the axon (Fig 1) [6]. The actin rings connect to each other via extended spectrin filaments [6–8]. The consensus in the field is that the periodic structure of the APMS contributes to the integrity and mechanical stability of the axon [5, 9–11].

A unique section of the axon called the axon initial segment (AIS) is usually located 20–60 μm from the soma and is the site of action potential initiation [12, 13]. The AIS also acts as a filter, separating membrane proteins and lipids between the axon and the somatodendritic neuronal subcompartments [14–18]. Researchers have proposed that the AIS regulates protein movement between the somatodendritic and axonal regions via a surface diffusion barrier and an intracellular traffic filter [14, 19]. This theory was confirmed by a single particle tracking (SPT) technique that measures the mobility of transmembrane proteins (TMPs) such as axonal cell-adhesion molecule L1 [18] and vesicle-associated membrane protein VAMP2 [17]. In addition, a recent study found that the motion of glycosylphosphatidylinositol-anchored green fluorescent protein (GPI-GFP) molecules in the AIS is confined within repetitive stripes with boundaries coinciding with the observed actin rings [20].

Currently, it is unknown how the AIS impedes the thermal motion of lipids and membrane proteins between the axon and soma. Two leading theories explain how the mobility of lipids and surface proteins is reduced in the AIS. Based on the “fence and picket” model [21], the reduced mobility is caused by steric hindrance between the membrane skeleton and the diffusing proteins, and is further enhanced by accumulation of membrane proteins tethered to membrane scaffolding molecules, such as ankyrin-G [15, 22, 23]. In a similar case, studies have shown that the membrane skeleton hinders lateral diffusion of band-3 proteins in the red blood cell membrane [24, 25]. The more recent “actin fence” model [14, 20] suggests that axon actin rings act as barriers and confine the motion of integral monotopic proteins (IMPs) on the outer lipid, within the ~190 nanometer area determined by actin rings.

It is possible that the “fence and picket” model, the “actin fence” model, or a combination of the two could explain diffusion in axonal membranes. However, current attempts to analyze experimental data based on the “actin fence” model are limited [14, 20], and no numerical studies have implemented the “fence and picket” model. More importantly, several aspects of APM protein diffusion cannot be directly answered by either model. For instance, given that actin is located intracellularly, it is not clear how the actin rings could interfere with lipids and IMPs of the outer leaflet of the phospholipid bilayer. In addition, we do not know whether diffusion of TMPs, lipids, and IMPs of the inner leaflet is influenced by the interactions of these macromolecules with the spectrin tetramers of the APMS. As the structure of the APMS is orthotropic, with a different geometry along the longitudinal versus the transverse direction, we might expect that longitudinal diffusion differs from transverse diffusion.

From a fundamental physics perspective, the type of macromolecule diffusion in a cellular membrane varies as it critically depends on the local membrane composition and the time scale. In some cases, the diffusion is normal, where the mean squared displacement (MSD) is proportional to time; in other cases, the diffusion is abnormal with MSD disproportional to time. Identifying different diffusion types may provide insight into membrane structure and its interactions with diffusing lipids and proteins. In the case of the APM, if membrane proteins cannot pass through the actin rings, the longitudinal MSD will eventually reach a constant value that is determined by the distance between consecutive actin rings, and the diffusion will become confined. When actin or actin-associated proteins interact with diffusing proteins or lipids via steric repulsion or transient association, steric hindrance or occasional trapping can occur. In this case, thermal motion can potentially become anomalous subdiffusion with the MSD proportional to *t*^*α*^, with (*α*<1). Depending on the time scale, diffusion may also vary from normal diffusion at small time scales, to transient anomalous diffusion at intermediate time scales, and to normal diffusion with a much lower diffusion coefficient at very large time scales [25, 26]. At small time scales, lipids and membrane proteins have not interacted yet with the boundary of the APMS corrals and the diffusion is defined as microscopic diffusion. At intermediate time scales, the interactions between diffusing particles and spectrin filaments, or between diffusing particles and mobile or tethered-to-the-APMS proteins will likely determine the type of transverse diffusion.

To discern the role of the periodic AMPS structure in lipid and protein diffusion of TMPs and IMPs of the outer and inner leaflets of the axon phospholipid bilayer, we developed a coarse-grain molecular dynamics (CGMD) model for the APM that includes the phospholipid bilayer, APMS, and axonal membrane proteins. Using our model, we (i) determined the role of actin rings in the diffusive motion of TMPs and IMPs of the inner leaflet within the areas between adjacent actin rings; (ii) showed that the association between spectrin filaments and lipids affects the circumferential (transverse) diffusion of TMPs and IMPs of the inner leaflet, but not the diffusion of the lipids and IMPs of the outer leaflet; (iii) determined that actin rings and spectrin filaments restrict the motion of membrane proteins along and around the circumference of the axon, respectively; and (iv) showed that the surface diffusion barrier in the AIS is formed as a result of both the accumulation of concentrated TMPs and the actin fence. Our findings help clarify how the APMS structure limits diffusion of proteins and lipids. Furthermore, it may provide critical insight on how the dysfunction of actin spectrin-associated proteins leads to neurological and neuropsychiatric disorders [27].

## Methods

### Computational model of the axon plasma membrane

We developed a particle-based mesoscale model of the APM of the neuronal axon that includes (1) the phospholipid bilayer as a double layer, including IMPs (in both inner and outer leaflets) and TMPs, and (2) the APMS. We followed an approach comparable to the one that we used to model the red blood cell (RBC) plasma membrane, which consists of structural elements similar to those in the neuronal APM [28–30]. Regarding the plasma membrane skeleton of the RBC and the axon, both consist of spectrin filaments connected at the actin junction, although they are arranged in a fundamentally different way. In the RBC membrane, the spectrin tetramers extend to an approximately equilibrium length and are connected at actin junctions to form a two-dimensional (2D) canonical hexagonal network that corresponds to an isotropic homogeneous 2D material [31]. In the neuronal APMS, actin is arranged in circular rings oriented along the circumference of the axon and connected to each other via spectrin filaments oriented along the longitudinal direction of the axon (Fig 1). The spectrin filaments extend to almost their contour length, meaning that they are under entropic tension. Because of this arrangement, the APMS behaves as an orthotropic material with different mechanical properties along the axon and perpendicular to the axon. We included here the model of the APMS for completeness. Its detailed description can be found in Zhang et al [11].

Evidence suggests that the connection between the APMS and the phospholipid bilayer is similar between the axon and the RBC membrane [6, 32, 33], where the spectrin network is tethered to the phospholipid bilayer via the ankyrin-band-3 complex and glycophorin. Specifically, in RBCs a β-spectrin dimer binds to ankyrin near its C terminus, which is located at approximately the middle area of the spectrin tetramer. Ankyrin then binds to the transmembrane ion channel band-3. In addition, actin junctional complexes bind to the phospholipid bilayer via glycophorin [34, 35]. In the AIS, the spectrin tetramers are most likely connected to ankyrin G, as in the RBC, and ankyrin G likely anchors the phospholipid bilayer to the APMS by binding to voltage-gated sodium (Na_v_) and potassium (K_v_) channels. Indeed, super-resolution microscopy experiments have shown that the Na_v_ channels exhibit a periodic ring-like distribution pattern that alternates with actin rings and co-localizes with ankyrin G [6, 8]. Although anchoring of the phospholipid bilayer to APMS at the middle area of spectrin tetramers via ankyrin G is likely, it is still unclear whether the phospholipid bilayer is also anchored at the actin rings. One possibility is that a TMP, playing the role of glycophorin in the RBC membrane, is connected directly or indirectly via an actin-associated protein to actin rings. We discuss the association between the APMS and the phospholipid bilayer in the axon further in the following section.

#### Periodic APMS structure

Studies have shown that actin rings in the APMS form a periodic structure of spectrin tetramers with a periodic distance of 180~190 nm. The tetramers connect such that the C termini of β-spectrin filaments alternate with actin rings [6, 8]. A spectrin tetramer consists of two identical heterodimers, each with 22 homologous triple helical repeats in the α-spectrin chain and 19 homologous triple helical repeats in the β-spectrin chain [34, 36]. To represent a spectrin tetramer, we used a chain of 41 particles connected by a spring potential $U^{SS}\left(r\right)=1/2k_{0}{\left(r‑r_{eq}^{SS}\right)}^{2}$, where the equilibrium distance between two consecutive particles is $r_{eq}^{SS}=L_{c}/40=5\;nm$ with *L*_*c*_≃200 *nm* being the contour length of a spectrin tetramer, and *k*_0_ determined in the following section. In addition, all spectrin particles interacted via the repulsive Lennard-Jones (L-J) potential (S1 Fig):

$$U_{rep}^{SS}(r_{\mathrm{ij}})=\{\begin{array}{cc} 4\varepsilon_{1}\left[{\left(\frac{2\sigma }{r_{\mathrm{ij}}}\right)}^{12}-{\left(\frac{2\sigma }{r_{\mathrm{ij}}}\right)}^{6}\right]+\varepsilon_{1} & r_{\mathrm{ij}}<R_{\mathrm{cut},\mathrm{LJ}}=r_{eq}^{SS}\\ 0 & r_{\mathrm{ij}}>R_{\mathrm{cut},\mathrm{LJ}}=r_{eq}^{SS} \end{array}$$ (1)

The equilibrium distance is $r_{eq}^{SS}=2\times 2^{1/6}\sigma =5\;nm$, which yields a unit length of *σ* = 2.227 nm. The value *ε*_1_ = 0.46*ε* gives an equilibrium curvature equal to the spring constant *k*_0_ in order to reduce the number of free parameters of the model. We chose *k*_0_ = 6.5*ε*/*σ*^2^ to be identical to the equilibrium curvature of the L-J potential used in the actin-spectrin interaction, with *ε* being the energy unit. We chose *R*_*cut*,*LJ*_ to be the equilibrium distance $r_{eq}^{SS}$ such that the L-J potential provides only the steric repulsive force between different spectrin particles. We measured the end-to-end distance of a single spectrin filament from thermal fluctuation of 10^6^ time steps after equilibration. The distances followed a Gaussian distribution with a mean value of ${\langle r_{ee}^{2}\rangle }^{1/2}=75.4\mathrm{nm}$ at *K*_*B*_*T*/*ε* = 0.22, where *K*_*B*_ was the Boltzmann’s constant and *T* was the temperature [11]. Assuming that the expression between the persistence length and the end-to-end distance for flexible filaments (*l*_*p*_<<*L*_*c*_) was given by ${\langle r_{ee}^{2}\rangle }^{1/2}≅\sqrt{2l_{p}L_{c}}$, and that the contour length of spectrin was approximately 200 nm [36, 37], we computed the persistence length of spectrin filaments to be 14.2 nm, which is close to reported experimental values of 10 nm [38] and 20 nm [39].

Next, we considered the connection between an end of a spectrin filament and the corresponding actin ring by a breakable L-J potential $U_{LJ}^{AS}(r_{ij})=4\varepsilon_{2}\left[{\left(8\sigma /r_{ij}\right)}^{12}-{\left(8\sigma /r_{ij}\right)}^{6}\right]+\varepsilon_{2}$. We chose the actin-spectrin association energy to be *ε*_2_ = 7.3*ε* which is approximately 0.86*eV*. This value was larger than the spectrin-actin-protein 4.1 complex association energy in normal RBCs, which was approximately 0.74*eV* [40]. We note that by setting the attraction force to zero beyond the capture distance of 2.5×8*σ*, the association between actin and spectrin could break and reform. We note that the actin-spectrin association energy in normal RBC membrane skeleton cannot maintain the integrity of the APMS when the distance between actin rings is 185 nm, in which case the spectrin filaments are under entropic tension. This means that the association energy of the spectrin-actin complex in the axon is most likely larger than that in normal RBCs. In the present model, the equilibrium distance between actin and spectrin is $2^{\frac{1}{6}}(8\sigma )≃20\mathrm{nm}$, resulting in an actin junction size of approximately 40 nm [41].

The mechanism underlying the formation of the actin rings in the periodic structure of the APMS is unclear. However, studies have shown that adducin co-localizes with actin rings and probably functions as a capping protein to stabilize actin filaments [6]. In addition to this plus-end capping protein, we expect that the minus ends of actin filaments are stabilized by other cross-linking proteins, such as tropomodulin in RBCs. Because the specific molecular structure of the actin rings and how the actin filaments are connected to form the ring-like structure in the axon is unknown, our CGMD model assumes a generic structure of the actin rings [11]. First, we chose an actin particle diameter of 35 nm, similar to the value used for the RBC membrane skeleton that comprises short actin oligomers (33±5nm) [42–46]. We used 39 particles to form an actin ring with a diameter of approximate 434 nm, which is consistent with experimental results for the unmyelinated neuronal axon [47]. We used a spring potential of $U^{AA}=1/2k_{A}{\left(r-r_{eq}^{AA}\right)}^{2}$ to connect two adjacent actin particles with an equilibrium distance of $r_{eq}^{AA}=35\;\mathrm{nm}$ and spring constant of *k*_*A*_ = 69.35*ε*/*σ*^2^ based on computational results in conjunction with an atomic force microscopy stiffness measurement of the axon membrane skeleton [11]. We used a repulsive L-J potential $U_{rep}^{AA}$ to simulate the steric interaction between actin particles with the same equilibrium distance as above. We also employed a finitely extensible nonlinear elastic (FENE) bending potential of $U_{b}=-\frac{1}{2}k_{b}\Delta \theta_{max}ln\left[1-{\left(\frac{\theta -\theta_{0}}{\Delta \theta_{max}}\right)}^{2}\right]$ to stabilize the circular shape of the actin rings, where *k*_*b*_ =3,500 *K*_*B*_*T* is the stiffness that directly regulates the bending rigidity of actin filaments. This *k*_*b*_ value resulted in a single actin filament bending rigidity of *κ*_*bend*_ = 7.1×10^−26^ *Nm*^2^, which is similar to a reported experimental rigidity of 7.3×10^−26^ *Nm*^2^ [48, 49]. The angle formed by three consecutive actin particles of the same ring was *θ*, with an equilibrium angle of $\theta_{0}=\frac{180{}^{\circ}\left(39-2\right)}{39}=170.77{}^{\circ}$ and a maximum allowed angle of Δ*θ*_*max*_ = 0.3*θ*_0_. We note that the combination of *k*_*b*_ and Δ*θ*_*max*_ determines the stiffness of the structure. In the case of small deformations, the value of Δ*θ*_*max*_ did not affect the behavior of actin rings near equilibrium. Because of this, we chose to use Δ*θ*_*max*_ = 0.3*θ*_0_, which allowed flexibility to the bending potential [11].

In addition to the actin and spectrin membrane skeleton, the microtubules and neurofilaments also play important roles in maintaining the mechanical structure of the axon. In our model, we considered that the microtubules interact with actin to keep the ring-ring distance at 185 nm. We introduced a FENE potential of $U_{mt}=-\frac{1}{2}k_{mt}\Delta d_{max}ln\left[1-{\left(\frac{d-d_{eq}^{RR}}{\Delta d_{max}}\right)}^{2}\right]$ to represent the effect of microtubules on maintaining the distance between two consecutive actin rings. The equilibrium distance between the centers of the two actin rings is $d_{eq}^{RR}=185\;\mathrm{nm}$, the maximum allowed deformation is $\Delta d_{max}=0.3\;d_{eq}^{RR}$, and the distance between two consecutive actin rings is *d*, which was calculated by measuring the mean value of the z-coordinate of particles that belong to the same ring. Finally, we determined $k_{mt}≃239\frac{K_{B}T}{\sigma }≃19,822\frac{K_{B}T}{d_{eq}^{RR}}$ at *T* = 300°*K* based on the longitudinal Young’s modulus of the axon *E*_*L*_≃10*kPa* [50]. Detailed information about the selection of parameters in this FENE potential can be found in [11].

#### Axonal membrane model

**Fig 2:**
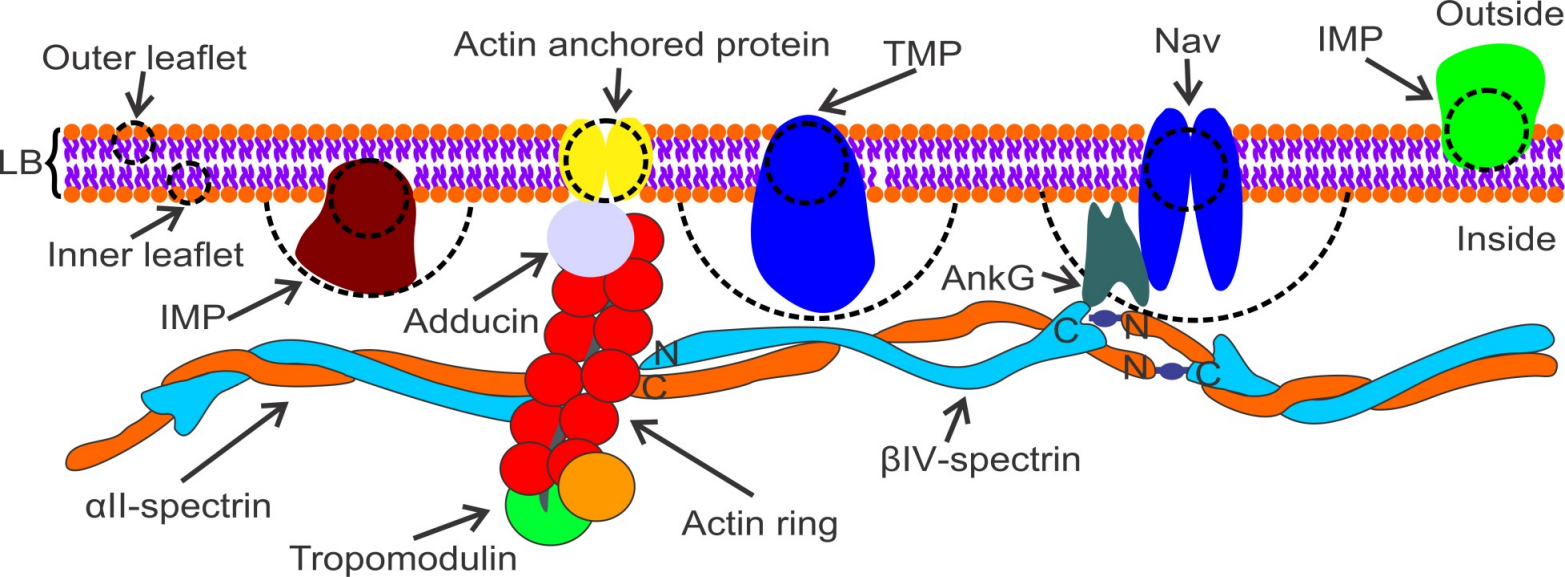
Illustration of the APM, which includes the APMS and the phospholipid bilayer. Ankyrin G binds to the 15^th^ repeat of β-spectrin near its carboxyl terminus and to Na_v_ in the axon. α-spectrin and β-spectrin filaments are connected at actin junctions. It is unknown how the phospholipid bilayer is anchored at the actin rings; we indicate a generic actin-associated protein that connects the actin rings to the phospholipid bilayer. Cluster of lipids in the individual layer are represented by a particle with a diameter of 2.5 nm, which is approximately the thickness of a single phospholipid bilayer. We chose the size of TMPs and IMPs of both the inner and outer leaflets to be 5 nm when they interact with other membrane proteins and lipids. However, the IMPs of the inner leaflet and TMPs have additional components that extend towards the cytosol, whereas the IMPs of the outer leaflet do not have inner extension regions. The effective size of the IMPs of the inner leaflet and the TMPs is considered to be 20 nm and 25 nm, respectively, when they interact with the APMS.

To model the phospholipid bilayer, we included a representation of two separate layers of lipids, IMPs of both inner and outer leaflets, and TMPs. Ankyrin-anchored TMPs, such as Na_v_ channels, are connected to spectrin filaments. As previously mentioned, although RBC actin junctions are anchored to the phospholipid bilayer by glycophorin, we still do not know how the actin rings are associated with the phospholipid bilayer in the neuronal axon. One possibility is that a TMP plays the role of a glycophorin of the RBC membrane, anchoring the phospholipid bilayer to actin rings. Fig 1A illustrates the computational model, where the red and gray colors denote a cluster of lipid molecules of inner and outer leaflets, respectively, with a diameter of 2.5 nm, which is approximately the thickness of a single phospholipid bilayer [31]. The yellow color particles represent actin-anchored proteins with a size of 5 nm. We note that when TMPs and IMPs interact with other membrane particles, the effective size in the membrane domain is 5 nm [51], which is approximately the thickness of the phospholipid bilayer. However, the IMPs of the inner leaflet (but not outer leaflet) and TMPs have additional components that extend towards the cytosol (Fig 2). We thus considered the effective size of IMPs of the inner leaflet and TMPs to be 20 nm and 25 nm, respectively [52, 53], when they interact with the APMS. For example, TMPs such as Na_v_ channels, which are connected to the spectrin filaments by ankyrin G, are located approximately at the middle of the spectrin tetramers and are represented as particles with a 25 nm diameter. This is also in agreement with the experimental finding that the effective radius of the cytoplasmic domain of the ankyrin complex connected to a Na_v_ channel is ~12.5 nm [54]. Other TMPs included in the computational model have no connection to the membrane skeleton.

The membrane particles have both translational and rotational degrees of freedom (**x**_*i*_,**n**_*i*_), where **x**_*i*_ and **n**_*i*_ denote position and orientation of the particle i, respectively. The direction vector **n**_*i*_ is normalized (|**n**_*i*_| = 1). The distance between two particles i and j is given by **x**_*ij*_ = **x**_*i*_−**x**_*j*_ with length *r*_*ij*_ = |**x**_*ij*_| and unit vector ${\hat{x}}_{ij}=x_{ij}/r_{ij}$. The pair potential between lipids and membrane proteins of the same layer is determined as *u*_*ij*,*mem*_(**n**_*i*_,**n**_*i*_,**x**_*ij*_) = *u*_*R*_(*r*_*ij*_)+*A*(*α*,*a*(**n**_*i*_,**n**_*j*_,**x**_*ij*_))*u*_*A*_(*r*_*ij*_), where *u*_*R*_(*r*_*ij*_) and *u*_*A*_(*r*_*ij*_) are the repulsive and attractive potential, respectively, given by:

$$U_{mem}(r_{\mathrm{ij}})=\{\begin{array}{c} u_{R}\left(r_{ij}\right)=k\varepsilon {\left(\left(R_{cut,mem}-r_{ij}\right)/\left(R_{cut,mem}-r_{eq}\right)\right)}^{8}\;\mathrm{for}\;r_{ij}<R_{cut,mem}\\ u_{A}\left(r_{ij}\right)=-2k\varepsilon {\left(\left(R_{cut,mem}-r_{ij}\right)/\left(R_{cut,mem}-r_{eq}\right)\right)}^{4}\;\mathrm{for}\;r_{ij}<R_{cut,mem}\\ u_{R}\left(r_{ij}\right)=u_{A}\left(r_{ij}\right)=0\;\mathrm{for}\;r_{ij}\geq R_{cut,mem} \end{array}$$ (2)

In this expression, we chose *R*_*cut*,*mem*_ to be 2.6*σ* in order to provide a fluid-like behavior of the membrane [28, 43] and *r*_*eq*_ as the equilibrium distance between two particles in the membrane domain, such as $r_{eq}^{LL}=2.5\;\mathrm{nm}$, $r_{eq}^{LG}=3.75\;\mathrm{nm}$, $r_{eq}^{LP}=3.75\;\mathrm{nm}$, $r_{eq}^{PP}=5\;\mathrm{nm}$, $r_{eq}^{GP}=5\;\mathrm{nm}$, and $r_{eq}^{GG}=5\;\mathrm{nm}$, where *L* represents lipids, *G* represents actin-anchored proteins, and *P* represents TMPs and IMPs. We selected *k* to be 1.2 for the interaction between lipid particles and 2.8 for the interaction between lipid particles and axonal membrane protein particles such as glycophorin and Na_v_ channels. *A*(*α*,*a*(**n**_*i*_,**n**_*j*_,**x**_*ij*_)) = 1+*α*(*a*(**n**_*i*_,**n**_*j*_,**x**_*ij*_)−1), where *α* = 1.55 is a linear amplification factor. The value of $a\left(n_{i},n_{j},x_{ij}\right)=\left(n_{i}\times {\hat{x}}_{ij}\right)•\left(n_{j}\times {\hat{x}}_{ij}\right)=n_{i}•n_{j}-\left(n_{i}•{\hat{x}}_{ij}\right)\left(n_{j}•{\hat{x}}_{ij}\right)$ varies from -1 to 1 and depends on both the distance and relative orientation. For example, *a* = −1 corresponds to the case when $\left(n_{i}↑↓n_{j}\right)⊥{\hat{x}}_{ij}$, and *a* = 1 corresponds to the case when $\left(n_{i}↑↑n_{j}\right)⊥{\hat{x}}_{ij}$ (S1 Fig). The strong orientation part of the membrane proteins is required to prevent the proteins to flip their orientation. Additionally, fixing the orientation allows for association of lipids with the membrane proteins. This maintains the IMPs and TMPs attached to their corresponding lipid leaflets. Details regarding the selection of the parameters in the lipid layer potential can be found in our previous publications [25, 28, 43]. The equations of motion include the translational and rotational components:

$m_{i}{\ddot{x}}_{i}=-\frac{\partial \left(V\right)}{\partial x_{i}}$ and ${\tilde{m}}_{i}{\ddot{n}}_{i}=-\frac{\partial \left(V\right)}{\partial n_{i}}+\left(\frac{\partial \left(V\right)}{\partial n_{i}}•n_{i}\right)n_{i}-{\tilde{m}}_{i}\left({\dot{n}}_{i}•{\dot{n}}_{i}\right)n_{i}$, where $V=\sum_{j=1}^{N}u_{ij,mem}$, ${\tilde{m}}_{i}$ is a pseudo-mass with units of energy x time^2^.

To model the interaction between the two lipid layers to form the phospholipid bilayer, we followed the approach proposed by Cooke and colleagues [55] and used the potential below.

$$U_{layer}(r_{\mathrm{ij}})=\{\begin{array}{l} u_{R}\left(r_{ij}\right)=4\varepsilon \left({\left(\sigma /r_{ij}\right)}^{12}-{\left(\sigma /r_{ij}\right)}^{6}\right)+(1-k)\varepsilon \;r_{ij}<R_{cut,layer}\\ u_{A}\left(r_{ij}\right)=-k\varepsilon \cos^{2}\left(\pi \left(r_{ij}-R_{cut,layer}\right)/2w_{c}\right)\;R_{cut,layer}\leq r_{ij}\leq R_{cut,layer}+w_{c}\\ \;0\;r_{ij}>R_{cut,layer}+w_{c} \end{array}$$ (3)

In particular, we used a repulsive L-J potential to reduce penetration between inner and outer layers. We modeled the attraction between the two lipid layers by using a potential that depends on cos^2^() that is more compliant than a corresponding L-J potential. We validated the selection of parameters *k* = 0.01, *w*_*c*_ = 1.3775*σ* by measuring the interfacial tension and bending rigidity of the phospholipid bilayer. Details related to these measurements and a list of the parameters used in the potential can be found in the Supporting Information (S2 and S3 Figs, S1 Table).

Finally, we modeled the association between the APMS and the phospholipid bilayer. In RBCs, band-3 proteins are connected to spectrin tetramers via ankyrin. Ankyrin then binds near the C terminus of one of the β-spectrin dimers at approximately the middle area of the spectrin tetramer. In addition, actin junctional complexes bind to the phospholipid bilayer via glycophorin and, thus, contribute to the anchoring of the phospholipid bilayer to the membrane skeleton [34, 35]. In the AIS, the spectrin tetramers are connected to ankyrin G as in RBCs. Then, ankyrin G anchors the APMS to the phospholipid bilayer by binding to Na_v_ channels. We assigned only one Na_v_ channel for each ankyrin molecule. The resulting Na_v_ channel density is approximately 150 channels per μm^2^, which lies within the range of 110 to 300 channels per μm^2^ measured in the AIS [56]. An ankyrin particle is connected to the 20^th^ particle of the spectrin filament by a spring potential $U^{SK}\left(r_{ij}\right)=1/2k_{0}{\left(r_{ij}-r_{eq}^{SK}\right)}^{2}$, where the equilibrium distance is $r_{eq}^{SK}=15\;\mathrm{nm}$. This distance corresponds to the radius of a spectrin particle (2.5 nm) and the effective radius of the cytoplasmic domain of the ankyrin complex connected to an Na_v_ channel (~12.5 nm) [54]. We used a similar spring potential of $U^{AG}\left(r_{ij}\right)=1/2k_{0}{\left(r_{ij}-r_{eq}^{AG}\right)}^{2}$ to represent the association between actin and actin-anchored proteins, where $r_{eq}^{AG}=20\;\mathrm{nm}$ is the equilibrium distance.

Because the binding force between spectrin filaments and the phospholipid bilayer in the axon is not known, we applied various strength levels in the attractive part of the L-J potential as follows:

$$U_{rep}^{LS}(r_{\mathrm{ij}})=\{\begin{array}{cc} 4n\varepsilon \left[{\left(\frac{1.5\sigma }{r_{\mathrm{ij}}}\right)}^{12}-{\left(\frac{1.5\sigma }{r_{\mathrm{ij}}}\right)}^{6}\right]+n\varepsilon & r_{\mathrm{ij}}\leq 3.75\sigma \\ 0 & r_{\mathrm{ij}}>3.75\sigma \end{array}$$ (4)

where *n* represents a parameter that adjusts the attractive energy. We used attraction between spectrin and lipid particles to investigate the corresponding effect on protein diffusion in the APM.

The system that we used in our simulation consists of 327,384 particles, corresponding to an axon with a length of approximately 555 nm. We implemented periodic boundary conditions at the two ends of the axon. We also note that the model of the APMS is periodic along the circumferential direction of the axon. Because of this, we considered that the circumferential length of the APMS was infinite when measuring its effect on the transverse diffusion of particles. We used the Beeman’s algorithm to integrate the equations of motion. The temperature of the system is controlled by the Nose-Hoover thermostat at *K*_*B*_*T*/*ε* = 0.22. The model is implemented in the *NVT* ensemble with a time scale of $t_{s}=\sqrt{m\sigma^{2}/\varepsilon }$ and time step of *dt* = 0.01*t*_*s*_. At the selected temperature, the proposed mesoscale implicit-solvent CGMD membrane model behaves as a 2D fluid where the lipids and axonal membrane proteins diffuse [25, 28, 43]. We first equilibrated the model for 3×10^6^ time steps and measured the diffusion of axonal membrane proteins during a period of 10^8^ time steps after equilibration. We performed the simulations on a high-performance computing cluster in the Department of Mechanical Engineering at the University of Connecticut and on the San Diego Supercomputer Center supported by the Extreme Science and Engineering Discovery Environment [57].

Below we describe limitations of the current model. First, it represents the lipid bilayer as two uniform single layer of particles. The coarse-grain lipid particle in each monolayer is treated as one type of lipid molecule. This was necessary in order to simulate the membrane thermal fluctuations and fluidity of the membrane at a much larger length and time scale. However, as the model lacks the specific lipid molecule structure or incorporation of lipid rafts, we cannot exclude the possibility that the interaction between different lipid molecule domains with membrane proteins might further regulate the diffusion of IMPs and TMPs in the membrane. Second, the mechanical structure of the axon is dictated not only by the actin and spectrin membrane skeleton but also by microtubules and neurofilaments. Here, we simulated the function of microtubules on maintaining the distance between two consecutive actin rings with a FENE potential without explicitly establishing the microtubules structures. However, as we did not simulate the role of mechanical stretch in axonal lipid and protein diffusion, we don’t think that the absence of precise simulation of microtubules or filaments would change our conclusions.

## Results

### Diffusion in the axon plasma membrane

In this study, we investigated the effects of the APMS and accumulation of TMPs on lipid and protein diffusion in the APM. Importantly, we considered the cylindrical shape of the axon when calculating the diffusion parameters [58]. To calculate the MSD as a function of time using numerical data, we took advantage of the fact that distances on a cylindrical surface are preserved during rolling unwrap. We first unwrapped the positions of the particles on the cylinder by rolling it on a 2D plane, and then calculated the MSD in the 2D plane (S4 Fig). We were able to neglect the radial displacements *dr* of membrane particles caused by thermal fluctuations as they were small compared to the radius *r* of the axon (< 5% of the axon radius). Consequently, a particle’s changed coordinates within the cylindrical coordinate system (*dr*,*rdθ*,*dz*) were transferred into a 2D plane surface as (*rdθ*,*dz*). Moreover, we measured the MSD (*r*^2^*dθ*^2^+*dz*^2^) on the cylindrical surface on the plane with coordinates *du* = *rdθ* and *dv* = *dz*. In several instances, we decoupled the thermal motion into longitudinal diffusion (1D diffusion along the axon) and transverse diffusion (1D diffusion along the axon’s circumference) as shown in S4 Fig. We reconstructed the overall 2D diffusive motion by combining the MSDs of the longitudinal and transverse components of diffusion.

### Actin rings as fences

**Fig 3:**
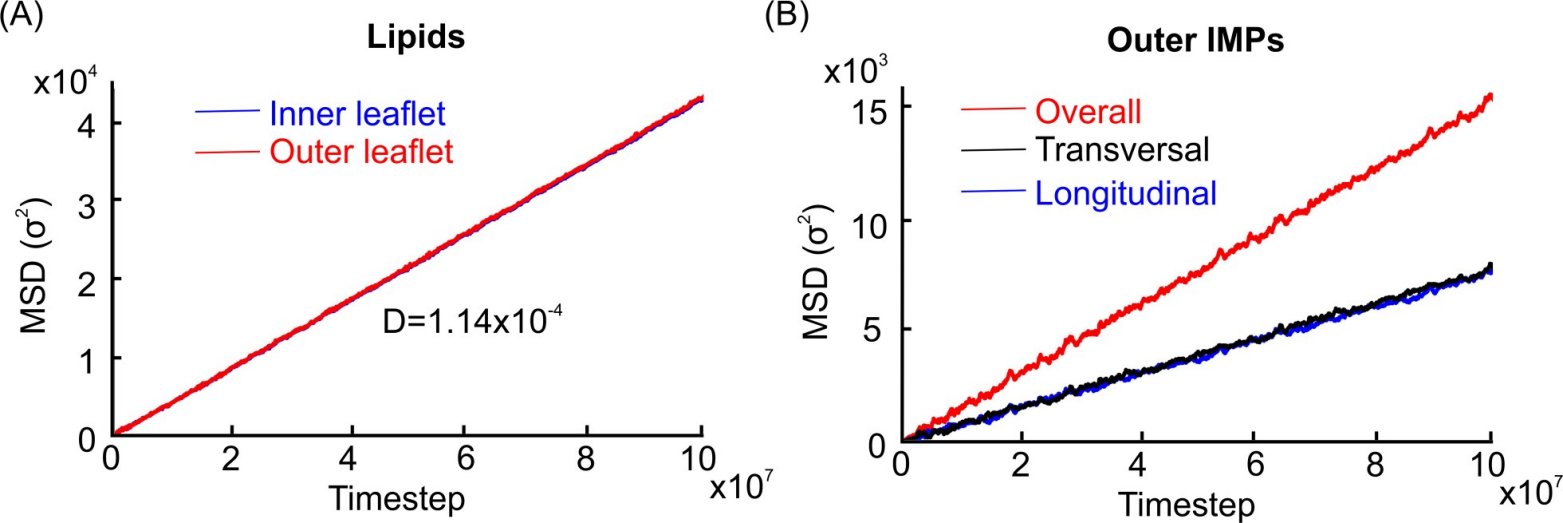
MSDs of lipids and IMPs of the outer leaflet. (A). 2D MSDs of lipids of inner and outer leaflets. (B). MSDs of outer leaflet IMPs in longitudinal and transversal directions. The overall MSD is the summation of longitudinal and transversal MSDs and represents surface diffusion.

We first established the time scale for the diffusion simulations. We measured diffusion of lipids and compared our simulations to the experimental results of Nakada and colleagues [16]. We found that the time dependence of MSD was similar for lipids in both the inner and outer leaflets (Fig 3A). This suggests that the diffusion of lipids in our model was not significantly affected by the APMS. Furthermore, as the dependence of MSD with respect to time was linear, the lipids underwent normal diffusion. We calculated the diffusion coefficient to be 1.14×10^−2^*σ*^2^/*t*_*s*_, where *σ* = 2.227 nm and is the time scale. By matching the diffusion coefficient of lipids *D*_*lipid*_ = 1.14×10^−2^*σ*^2^/*t*_*s*_ with the experimentally measured diffusion coefficient of ~0.3 μm^2^/s [16], we determined that *t*_*s*_ = 1.82×10^−7^s. We note that the membrane viscosity can be found by the Stokes-Einstein equation *η* = *K*_*B*_*T*/6*πD*_*lipid*_*r*_*P*_, where *r*_*P*_ is the radius of the coarse grain lipid particle. Using the determined time scale, we found that the corresponding membrane viscosity is 0.6*N*⋅*s*/*m*^2^, which approximates the reported experimental data [59]. We further validated this result by calculating the diffusion coefficient of the IMPs of the outer leaflet. By fitting the MSD for the IMPs of the outer leaflet with a linear function of time (Fig 3B), we determined that the diffusion coefficient was *D*_*outer*_ = 3.84×10^−3^*σ*^2^/*t*_*s*_ = 0.11 μm^2^/s. This result is similar to the experimentally measured diffusion coefficient value for GPI-GFP in the AIS, before the establishment of diffusion strips, and in the distal axon, which lacks diffusion strips [20]. After establishing a time scale that is consistent with experimental results, we determined the conditions in which actin rings could act as “fences” for diffusing outer leaflet, inner leaflet, and transmembrane proteins.

#### Diffusion of IMPs in the outer leaflet

Albrecht and colleagues showed that diffusion of IMPs of the outer leaflet is confined to rings that co-localize with the actin rings of the AIS, which possibly act as “fences” [20]. This begs the question: how is the motion of IMPs in the outer leaflet restricted by the sub-membrane cytoskeleton and, in particular, by the actin rings? We derived trajectories for the outer leaflet IMPs in an effort to understand this paradoxical observation.

Our initial results did not show any obvious actin boundaries for the IMPs of the outer leaflet, whereas the MSDs of the longitudinal and transverse components of diffusion were both linear functions of time (Fig 3B and S5 Fig). We next considered several other explanations as to how actin might hinder the outer leaflet diffusion. One possibility was that an increased association between actin particles and diffusing IMPs of the outer leaflet—independent of actin-associated proteins—could cause boundary formation and restrict IMP diffusion. We hypothesized that the association between actin and IMPs was restricted to only one protein per actin particle. We showed that actin particles can transiently associate with diffusing protein particles, but this association did not lead to the formation of well-defined boundaries for diffusing IMPs (S6 Fig).

**Fig 4:**
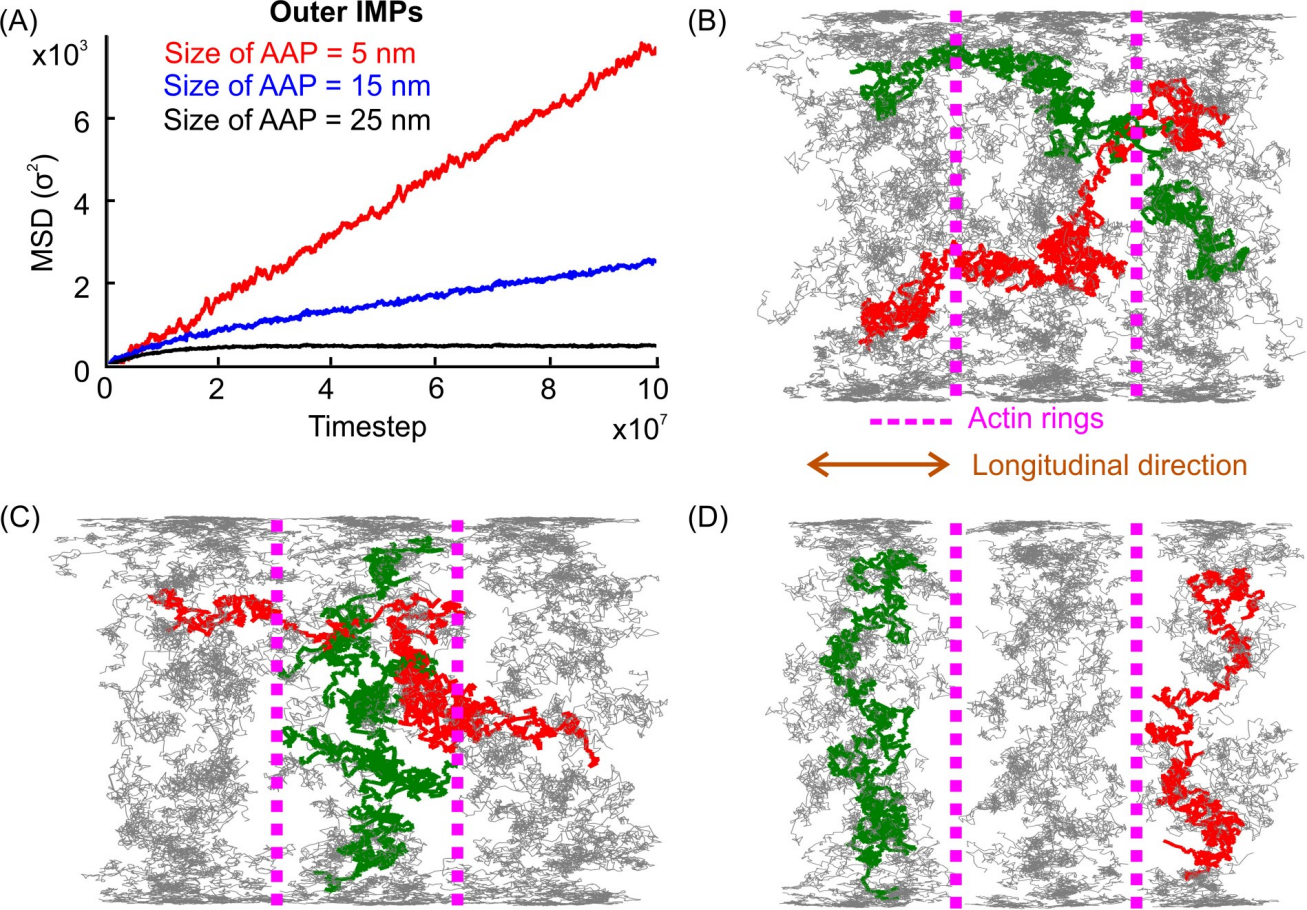
MSDs and trajectories of the outer leaflet IMPs with actin-anchored proteins (AAPs) of different sizes. (A). Longitudinal MSDs of IMPs in the outer leaflet with three sizes of AAPs. Panels (B), (C), and (D) show the trajectories of 5 nm, 15 nm, and 25 nm AAPs, respectively.

Finally, we tested an alternative explanation: that actin rings were not hampering the mobility of IMPs, but rather this hindrance was caused by proteins that form actin junction complexes, more specifically, proteins that associate the actin rings with the phospholipid bilayer. In this model, actin-associated proteins are obstacles for diffusing IMPs, impeding their mobility [14]. To test this possibility, we performed simulations with actin-anchored proteins of different sizes (5 nm, 15 nm, and 25 nm). Fig 4 shows the trajectories of the smallest particles (5 nm). We did not find any obvious boundaries that restricted the motion of IMPs in the outer leaflet. However, as the size of the particles representing actin-anchoring proteins grew to 15 nm, we recorded a decrease in the frequency of actin ring crossing. We observed clear actin boundaries and thus confined diffusion along the longitudinal directions when the diameter of the actin-anchoring proteins was 25 nm. As expected, the longitudinal motion of the outer leaflet IMPs also changed from free to confined diffusion when the particle diameter was set at 25 nm. Therefore, our simulations show that actin-associated proteins are necessary for restricting the diffusive motion of the outer leaflet IMPs.

#### Diffusion of IMPs in the inner leaflet

After establishing the conditions for which actin rings could restrict diffusion of IMPs in the outer leaflet between two adjacent rings, we determined the role of actin rings on the diffusion of inner-leaflet TMPs and IMPs along the axon’s longitudinal direction. To our knowledge, no available experimental results to which we could compare our numerical result exists. Thus, this simulation provides a framework for future experiments. We expect the actin rings to interfere with longitudinal diffusion. By contrast, the spectrin network that connects the actin rings might interfere with transverse diffusion. We therefore only discuss longitudinal diffusion in this section. We discuss transverse diffusion in the next section.

**Fig 5:**
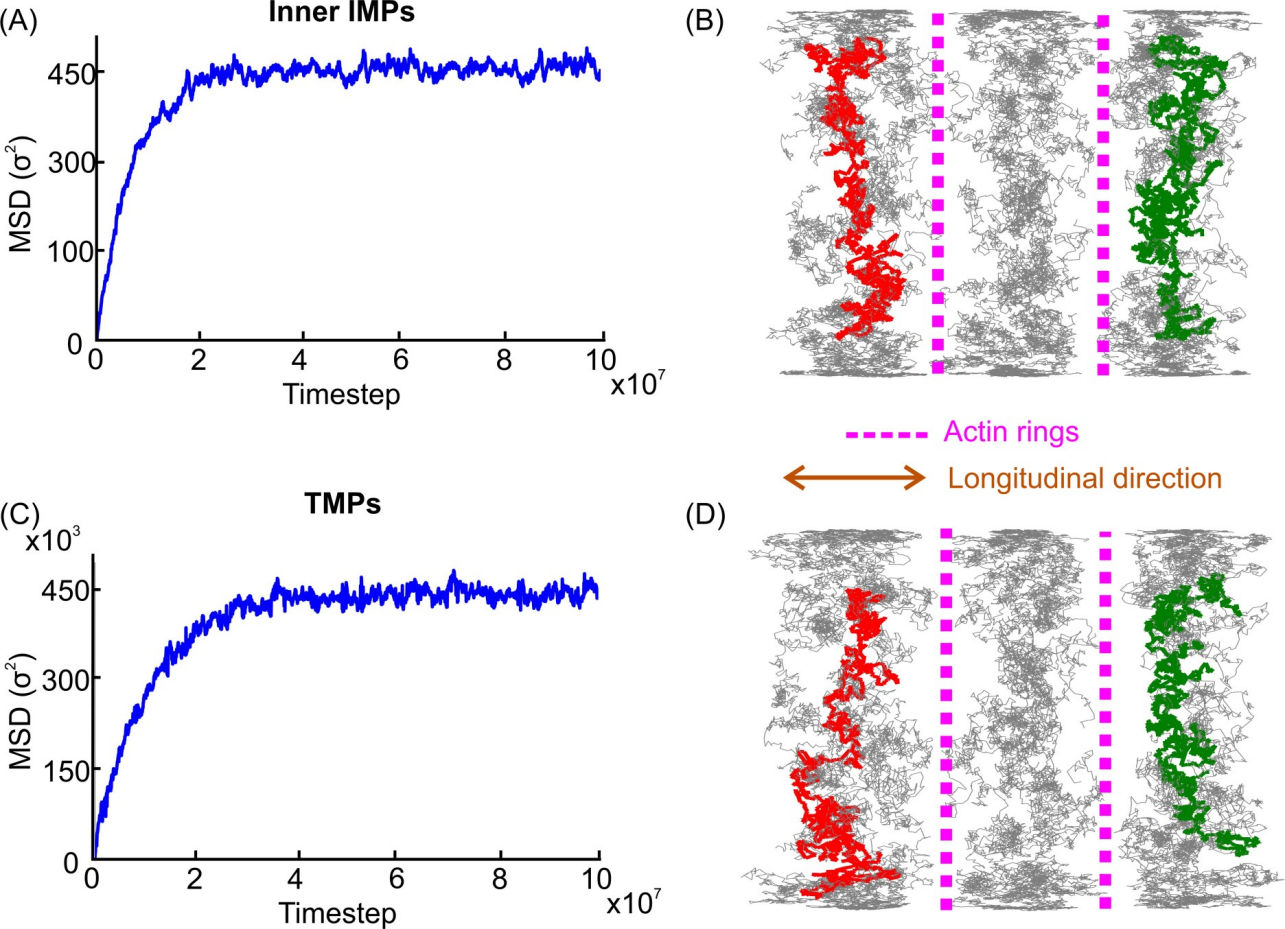
MSDs and trajectories of IMPs in the inner leaflet and TMPs. (A). Longitudinal MSD of IMPs in the inner leaflet. (B). Trajectories of IMPs in the inner leaflet. (C). Longitudinal MSD of TMPs. (D). Trajectories of TMPs.

With regards to longitudinal diffusion, we found that the inner leaflet TMPs’ and IMPs’ motions could be described as confined diffusion (Fig 5) due to the restriction imposed by the actin rings [26, 60–62]. The MSD of a completely confined diffusion is given by the expression $MSD(t)=L^{2}/6(1-e^{-12D_{micro}t/L^{2}})$, where *L* is the characteristic length and *D*_*micro*_ is the microscopic diffusion coefficient [60]. By fitting the numerical results to the aforementioned expression, we found that, in the case of TMPs, the characteristic length was *L*≈48.5*σ* = 108 nm and the microscopic diffusion coefficient was $D_{micro,TMP}^{Longitudinal}=1.53\times 10^{-3}\sigma^{2}/t_{s}=0.04\;{\mu m}^{2}/s$ (S2 Table and S7 Fig). By including the steric distance of 30*nm* between a TMP and the actin particle to both sides of an axon’s cylindrical stripe, we obtained a stripe width of 168 nm, which is close to the 185 nm distance between the two actin rings employed in our model. This result justifies that *L* can be considered a measure of the average maximum distance covered by a TMP along the axon. For IMPs of the inner leaflet, we found that the characteristic length is *L*≈52*σ* = 115.8 nm and the microscopic diffusion coefficient is $D_{micro,inner}^{Longitudinal}=3.31\times 10^{-3}\sigma^{2}/t_{s}=0.09\;{\mu m}^{2}/s$. As expected, this diffusion coefficient value was similar to the value obtained for IMPs of the outer leaflet. The diffusion coefficients differed between TMPs and IMPs due to their different radii, which caused TMPs to interact with particles from both lipid layers and IMPs to interact with particles from only one lipid layer.

In summary, we found that actin rings could limit longitudinal diffusion of inner but not of outer leaflet lipids and proteins. Additional actin-associated proteins that anchor the phospholipid bilayer to actin rings are necessary to limit the diffusion of outer leaflet lipids and proteins.

### Role of spectrin filaments as permeable “fences”

Next, we investigated the role of spectrin filaments on the diffusion of axonal membrane proteins. Ample evidence indicates that spectrin filaments are oriented along the axon, and the N terminus of βIV spectrin is connected to the actin rings. We previously showed that the spectrin filaments are under entropic tension and the periodic actin/spectrin arrangement provides the axon with mechanical resistance and flexibility [11]. As discussed in the previous section, actin rings wrap along the axon’s circumferential direction and restrict the motion of TMPs and IMPs of the inner leaflet in the longitudinal direction. Here, we examined whether the spectrin filaments exert a similar effect on the transverse diffusion of axonal IMPs of inner and outer leaflets.

#### Transverse diffusion of IMPs of the outer leaflet for different spectrin-phospholipid bilayer associations

For these simulations, we assumed only a steric repulsion between spectrin filaments and the phospholipid bilayer (*n* = 0 in Eq 4). The MSDs of the outer leaflet IMPs showed a linear dependence on time (S8 Fig). The corresponding transverse diffusion coefficients $D_{lipid}^{Transverse}=1.15\times 10^{-2}\sigma^{2}/t_{s}$ and $D_{outer}^{Transverse}=3.82\times 10^{-3}\sigma^{2}/t_{s}$ were similar to the longitudinal diffusion coefficient (Fig 3). This means that the IMPs of the outer leaflet undergo normal diffusion in both the longitudinal and transverse directions, and the corresponding diffusion is not affected by the sub-membrane spectrin filaments. This result is not surprising because the IMPs of the outer leaflet do not directly interact with the spectrin filaments.

#### Transverse diffusion of TMPs and IMPs of the inner leaflet for different spectrin-phospholipid bilayer associations

The diffusion of TMPs and IMPs of the inner leaflet are likely abnormal given that TMPs and IMPs directly interact with the APMS. This type of diffusion depends on the interactions between spectrin filaments and diffusing particles. If the lipid layer and the spectrin particles have a strong attraction, the spectrin filaments remain very close or even attached to the cytoplasmic lipid layer and act as impenetrable reflecting barriers. In this case, we expect that the transverse diffusion would be confined to the cortex corals formed by the consecutive spectrin filaments and the corresponding portions of the actin rings (Fig 1). If the lipids and spectrin particles are only weakly attracted to one another, we expect the oscillating spectrin filaments to form transient barriers with the oscillating phospholipid bilayer, which could then allow diffusing particles to occasionally escape from one compartment to the other. This motion is called hop diffusion. The steric hindrance effect we observed between spectrin filaments and TMPs and IMPs depends on the characteristic time scales of membrane oscillations, spectrin oscillations, and diffusion. The characteristic conformation time for spectrin filaments is approximately 100 μs [28, 63]; by contrast, the characteristic period of plasma membrane oscillations is approximately 500 μs [64, 65]. The free-diffusion coefficient for TMPs and IMPs in phospholipid bilayers is 5.3×10^−9^*cm*^2^*s*^−1^ [66]. Based on this result, we calculated that a membrane protein takes approximately 200 μs to travel approximately 10 nm, which is the distance the protein must cover to clear a spectrin barrier. Given that the aforementioned characteristic time scales are of the same order of magnitude, TMPs and IMPs could cross the barriers formed by the spectrin filaments if there is no association between spectrin and the phospholipid bilayer.

**Fig 6:**
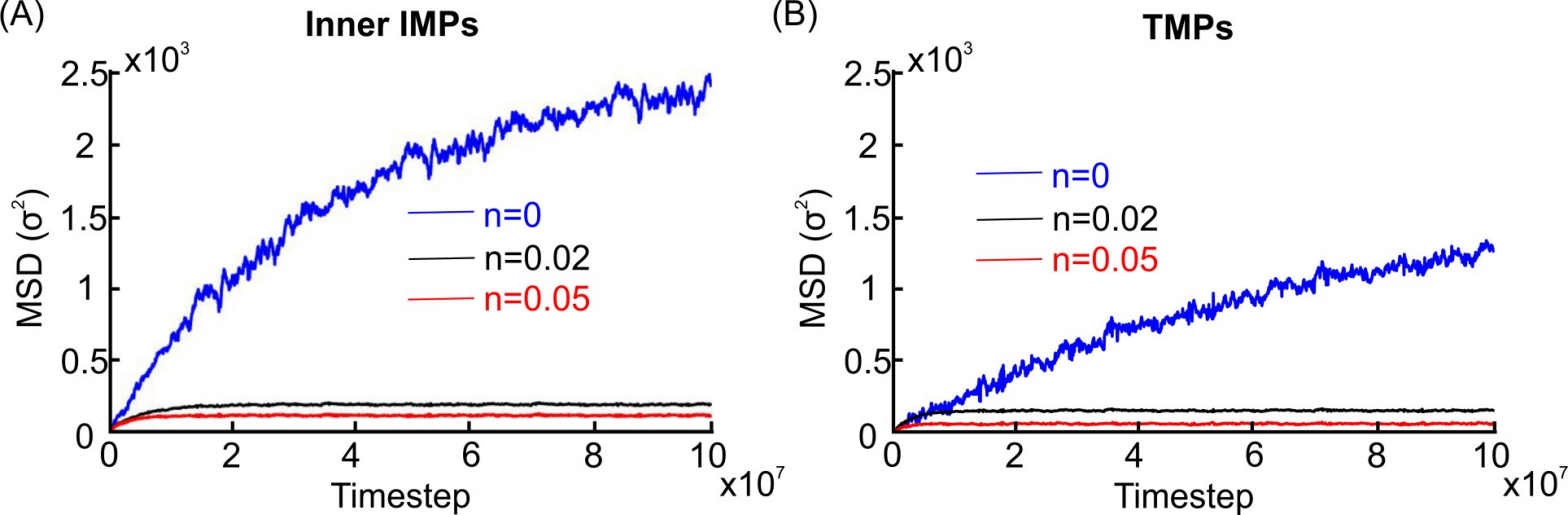
MSDs of the inner leaflet IMPs and TMPs along the axon’s circumferential direction. (A). Transverse MSD of the inner leaflet IMPs at different spectrin-lipid associations. (B). Transverse MSD of TMPs at different spectrin-lipid associations.

When we examined the diffusion of TMPs and IMPs of the inner leaflet, the MSDs showed a nonlinear dependence on time when the spectrin filaments and lipid particles did not associate (blue lines in Fig 6). We can explain the sub-diffusion behavior through two types of diffusion: (i) anomalous diffusion and (ii) confined diffusion at short time scales superimposed on linear, slow, normal diffusion at longer time scales.

Theoretically, anomalous sub-diffusion is generated by an infinite hierarchy of binding sites and barriers that hinder diffusion, and its MSD is proportional to ~t^a^, where a<1 [67, 68]. In cells, binding sites and barriers have a finite hierarchy and, consequently, the diffusion could be transient anomalous sub-diffusion for relatively short times and normal diffusion for longer times. Our model of the APMS, however, has an infinite number of spectrin filaments, as we used periodic boundary conditions. Therefore, we would not exclude the possibility of anomalous transverse diffusion across all time scales except for the very small time scale related to ballistic motion.

If the model of confined transverse diffusion is used at relatively short times and normal slow transverse diffusion is used at long times, then the MSD can be described by the expression $MSD(t)=L^{2}/6(1-e^{-12D_{micro}t/L^{2}})+2D_{macro}t$ [60]. In particular, we expect two diffusion coefficients: one that is valid at time scales that are significantly smaller than the characteristic crossing time (*D*_*micro*_), and one that is valid at much larger time scales (*D*_*macro*_). In RBCs, the experimental results revealed that band-3 proteins undergo hop diffusion. It was hypothesized that this is due to the cytoskeleton remodeling and binding forces between spectrin and lipids promote band-3 hop diffusion [26, 66]. Our simulations for the normal and defective RBC membrane showed that band-3 diffusion type is controlled by the connectivity of the membrane skeleton and the association between the phospholipid bilayer and membrane skeleton [25].

In order to clarify which model best explains our numerical results, we employed the expressions for both anomalous diffusion *MSD*(*t*) = *Ct*^*α*^ and confined-hop diffusion $MSD(t)=L^{2}/6(1-e^{-12D_{micro}t/L^{2}})+2D_{macro}t$ to fit the MSDs of TMPs and IMPs of the inner leaflet along the axon’s circumferential direction. The value of the residual’s squared norm served as our criterion for best fit (S9 Fig). The results show that confined-hop diffusion with long-time normal diffusion is a better approximation than anomalous diffusion of TMPs and IMPs of the inner leaflet. By employing the confined-diffusion model, we obtained $D_{micro,inner}^{Transverse}=3.38\times 10^{-3}\sigma^{2}/t_{s}=0.09\;{\mu m}^{2}/s$ and $D_{micro,TMP}^{Transverse}=1.26\times 10^{-3}\sigma^{2}/t_{s}=0.03\;{\mu m}^{2}/s$, which resemble the values of $D_{micro,inner}^{Longitudinal}$ and $D_{micro,TMP}^{Longitudinal}$, respectively as shown in S3 Table. We calculated the macroscopic diffusion coefficients of TMPs and IMPs of the inner leaflet along the axon’s circumferential direction to be $D_{macro,TMP}^{Transverse}=8.97\times 10^{-5}\sigma^{2}/t_{s}$ and $D_{macro,inner}^{Transverse}=1.86\times 10^{-4}\sigma^{2}/t_{s}$, respectively. We note that these values are 10 to 20 times smaller than the corresponding microscopic diffusion coefficients.

Next, we investigated transverse diffusion for different levels of attraction between spectrin and lipids. This is particularly important because βIV spectrin has a pleckstrin domain that allows spectrin to interact with the phospholipid bilayer. First, we found that the transverse diffusion of the IMPs of the outer leaflet was not affected by the spectrin filaments even when spectrin and lipid particles were strongly associated (n = 0.02 or 0.05 in Eq 4). Specifically, we observed an approximately linear dependence of MSDs with respect to time (S8 Fig) that produced a diffusion coefficient of *D*_*lipid*,*n* = 0.02_ = 1.09×10^−2^*σ*^2^/*t*_*s*_, *D*_*lipid*,*n* = 0.05_ = 1.07×10^−2^*σ*^2^/*t*_*s*_ for lipids and *D*_*outer*,*n* = 0.02_ = 3.77×10^−2^*σ*^2^/*t*_*s*_, *D*_*outer*,*n* = 0.05_ = 3.65×10^−2^*σ*^2^/*t*_*s*_ for IMPs of the outer leaflet. These values closely resembled the diffusion coefficients (*D*_*lipid*,*n* = 0_ = 1.14×10^−2^*σ*^2^/*t*_*s*_, *D*_*outer*,*n* = 0_ = 3.84×10^−3^*σ*^2^/*t*_*s*_) for free lipids and free IMPs of the outer leaflet, respectively, that move in a flat phospholipid bilayer without spectrin barriers.

By contrast, the MSDs of TMPs and IMPs of the inner leaflet were characterized by confined diffusion behavior (black and red lines; Fig 6). By fitting the numerical data to the expression for the MSD displacement in confined diffusion, we extracted their corresponding microscopic diffusion coefficients $D_{micro,inner}^{Transverse}=2.94\times 10^{-3}\sigma^{2}/t_{s}=0.08\;{\mu m}^{2}/s$, $D_{micro,TMP}^{Transverse}=1.34\times 10^{-3}\sigma^{2}/t_{s}=0.03\;{\mu m}^{2}/s$ at n = 0.02, $D_{micro,inner}^{Transverse}=3.04\times 10^{-3}\sigma^{2}/t_{s}=0.08\;{\mu m}^{2}/s$, and $D_{micro,TMP}^{Transverse}=1.44\times 10^{-3}\sigma^{2}/t_{s}=0.04\;{\mu m}^{2}/s$ at n = 0.05. The microscopic diffusion coefficient values in the transverse direction resembled the values observed for the confined longitudinal diffusion and the normal diffusion of the corresponding proteins. For the inner leaflet IMPs, the characteristic length was *L*≈5.91*σ*= 13.16 nm at n = 0.02 and *L*≈5.09*σ*= 11.34 nm at n = 0.05. In addition, we calculated the characteristic length of TMPs to be *L*≈4.15*σ*= 9.24 nm at n = 0.02 and *L*≈3.22*σ*= 7.17 nm at n = 0.05. By adding the corresponding steric distance to a spectrin particle, we obtained a distance that was close to the periodic circumferential distance between the spectrin filaments, which was approximately 35 nm.

We conclude that repulsive interaction between the axonal membrane proteins and the spectrin filaments results in confined diffusion of TMPs and IMPs of the inner leaflet when spectrin filaments and lipids are strongly associated. When there is no attraction between the spectrin and lipid particles, the oscillations of lipid layer and spectrin filaments hinder the motion of axonal membrane proteins but do not completely prohibit crossing of the spectrin barriers. Thus, TMPs and IMPs of the inner leaflet can hop over the spectrin corrals, which leads to normal diffusion at a larger time scale. However, this process is characterized by a smaller macroscopic diffusion coefficient compared to the microscopic one. Therefore, MSDs at larger time scales account for the inter-compartmental hop diffusion between spectrin filaments in the circumferential direction.

### Effect of accumulation of TMPs on the diffusion of lipids and membrane proteins in the APM of the AIS

In neurons that have matured past ten days *in vitro* (DIV), the AIS acts as a diffusion barrier and completely halts diffusion while the structure of the APMS remains essentially the same. By contrast, among neurons that are no older than a week (< DIV 6), proteins within the AIS are diffusive. It is possible that the lack of diffusion in the AIS at the later developmental time points is due to accumulation of ankyrin-binding TMPs in the AIS, such as Na and potassium channels [69, 70]. We have demonstrated that the APMS hinders, but does not completely stop, the thermal motion of axonal membrane proteins. This result is similar to an earlier experimental finding in cochlea outer hair cells that showed cytoskeletal structures alone constraint but do not completely prevent lateral mobility of membrane proteins [71]. We therefore hypothesized that accumulation of TMPs in the AIS plays an important role in ceasing diffusion of APM proteins.

**Fig 7:**
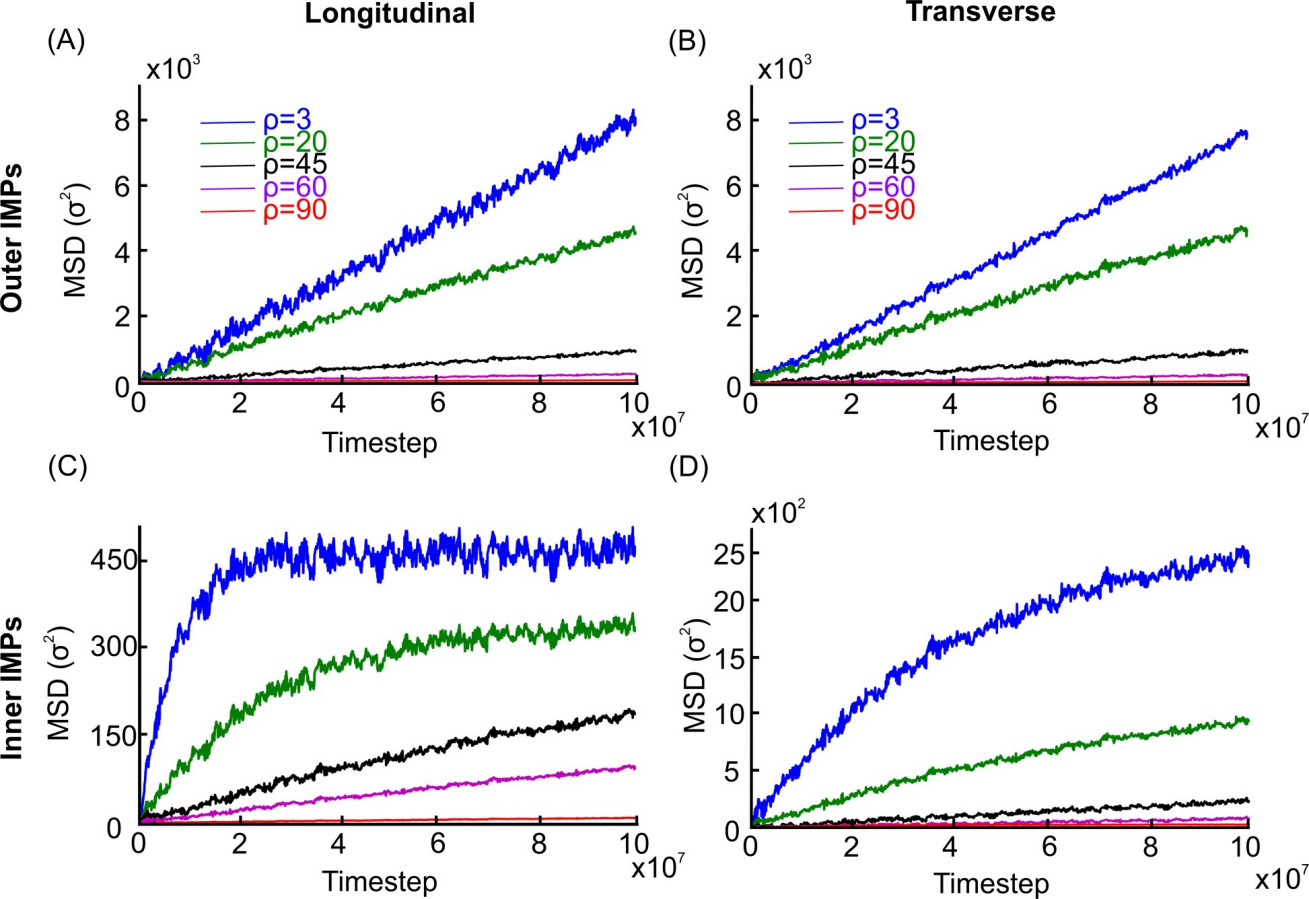
One-dimensional MSDs of IMPs at different accumulation levels of anchored TMP. (A). Longitudinal and (B). Transverse MSDs of IMPs of the outer leaflet at 3, 20, 45, 60 and 90 pprc. (C). Longitudinal and (D). Transverse MSDs of IMPs of the inner leaflet at 3, 20, 45, 60 and 90 pprc. The percentages of surface area coverage are 0.87%, 5.83%, 13.11%, 17.48%, and 26.22%, respectively.

We considered different accumulation levels of TMPs that were anchored to the APMS with a limited motion range as is typically the case with ion channels such as voltage-gated sodium (Na_v_) and potassium (K_v_) channels (i.e KCNQ2). We attached the TMPs particles at random points of the middle surface of the lipid bilayer via a spring with stiffness *k*_0_ = 6.5*ε*/*σ*^2^. This resulted to an average radius of gyration *R*_*g*_≃4.83 *nm* of the anchored TMPs in the APM comparable to their diameter. We then created different environments by increasing the initial surface accumulation of TMPs from three particles per rectangular corral (ρ = 3 pprc) to 20, 45, 60, and 90 (S10 Fig). These surface densities of TMPs corresponds to surface area coverages of 0.87%, 5.83%, 13.11%, 17.48%, and 26.22%, respectively. We first measured the MSDs of IMPs of the outer leaflet at the axon’s longitudinal and transverse directions at different TMP densities, with no attraction between spectrin filaments and lipid particles (n = 0 in Eq 4). We observed that increased accumulation of TMPs did not noticeably and for the observed time scale alter the diffusion type of IMPs of the outer leaflet, which remained normal (Fig 7A and 7B). However, it caused a reduction in both the longitudinal and transverse diffusion coefficients and the total corresponding diffusion coefficients from *D*_*outer*,3 *pprc*_ = 3.84×10^−3^*σ*^2^/*t*_*s*_ for ρ = 3 pprc to *D*_*outer*,20 *pprc*_ = 2.36×10^−3^*σ*^2^/*t*_*s*_ for ρ = 20 pprc, *D*_*outer*,45 *pprc*_ = 7.43×10^−4^*σ*^2^/*t*_*s*_ for ρ = 45 pprc, *D*_*outer*,60 *pprc*_ = 1.41×10^−4^*σ*^2^/*t*_*s*_ for ρ = 60 pprc, and *D*_*outer*,90 *pprc*_ = 2.24×10^−5^*σ*^2^/*t*_*s*_ for ρ = 90 pprc, as shown in S4 Table. From the result it is clear that at ρ = 90 pprc (~26% surface coverage) the diffusive motion of IMPs of the outer leaflet almost ceased since the corresponding diffusion coefficient *D*_*outer*,90 *pprc*_ for 90 pprc is more than 500 times smaller than the diffusion coefficient *D*_*lipid*_ = 1.14 × 10^−2^*σ*^2^/*t*_*s*_ of particles that represent lipids. It is noted however that this reduction in diffusivity cannot cause the formation of stripes. Similarly with the IMPs of the outer leaflet, diffusion of particles representing lipids remained normal but the diffusion coefficient reduced as the accumulation of TMPs increased and at ρ = 90 pprc diffusion practically ceased (S11 Fig and S4 Table).

Next, we measured the MSDs of IMPs of the inner leaflet at the axon’s longitudinal and transverse directions at different TMP densities, with no attraction between spectrin filaments and lipid particles (n = 0 in Eq 4). As discussed previously, IMPs of the inner leaflet underwent confined longitudinal diffusion. This is apparent in Fig 7C for ρ = 3 pprc and ρ = 20 pprc, where MSDs almost reached their maximum value for the observed number of time steps. For ρ = 45 pprc, ρ = 60 pprc, and ρ = 90 pprc, although the longitudinal diffusion was eventually confined, the required number of time steps for the MSDs to reach their maximum value went beyond the observed time period. For example, if we used the characteristic length *L* = 115.8*nm*, computed in the case of ρ = 3 pprc for IMPs of the inner leaflet, we found that when ρ = 45 pprc, approximately 8×10^7^ more time steps were required to reach the maximum MSD value. This meant that at ρ = 45 pprc (13.11% area coverage), the motion of IMPs of the inner leaflet was significantly hindered and the number of time steps in our simulation was not large enough to see the effect of corrals.

The effect of the accumulation of TMPs was clearly reflected on the value of the microscopic diffusion coefficient, which corresponds to the slope of the linear portion of the MSD graph at small time scale. By fitting the corresponding MSDs with the expression of confined-hop diffusion, we found that as the density ρ increased from 3 to 20, 45, 60, and 90 pprc, the microscopic diffusion coefficient for the IMPs of the inner leaflet decreased from $D_{inner,3\;pprc}^{Longitudinal}=3.26\times 10^{-3}\sigma^{2}/t_{s}$ to $D_{inner,20\;pprc}^{Longitudinal}=2.04\times 10^{-3}\sigma^{2}/t_{s}$ to $D_{inner,45\;pprc}^{Longitudinal}=1.65\times 10^{-4}\sigma^{2}/t_{s}$ and $D_{inner,60\;pprc}^{Longitudinal}=9.87\times 10^{-5}\sigma^{2}/t_{s}$ to $D_{inner,90\;pprc}^{Longitudinal}=2.63\times 10^{-5}\sigma^{2}/t_{s}$ respectively (Fig 7C and S5 Table). In transverse diffusion, accumulation of TMPs once again failed to change the nature of diffusion and merely reduced the micro-diffusion coefficient. As discussed above, transverse diffusion can be described as confined hop diffusion. For the values ρ = 3, 20, 45, 60, and 90 pprc the micro-diffusion coefficients for the IMPs of the inner leaflet decreased from $D_{inner,3\;pprc}^{Transverse}=3.28\times 10^{-3}\sigma^{2}/t_{s}$ to $D_{inner,20\;pprc}^{Transverse}=2.13\times 10^{-3}\sigma^{2}/t_{s}$ to $D_{inner,45\;pprc}^{Transverse}=1.43\times 10^{-4}\sigma^{2}/t_{s}$, and $D_{inner,60\;pprc}^{Transverse}=8.76\times 10^{-5}\sigma^{2}/t_{s}$ to $D_{inner,90\;pprc}^{Transverse}=2.66\times 10^{-5}\sigma^{2}/t_{s}$ (Fig 7D and S5 Table).

For completeness, we also investigated how accumulation of TMPs, which are not anchored to the APMS but that can thermally diffuse, affects the diffusive motion of IMPs of the inner and outer leaflet. We previously considered the effect of APMS, when the density of TMPs between the actin rings was set to ρ = 3 pprc, which was a very low density compared to the density of membrane proteins in the axons of mature neurons (no accumulation of TMPs; S10 Fig) [69, 70]. To study the effect of the accumulation of TMPs, we again created different environments by increasing the initial surface density of TMPs from ρ = 3 to 20, 45, 60, and 90 pprc. We found that the TMPs behave similarly to IMPs of the inner leaflet. The longitudinal diffusion was confined and the transverse diffusion was confined hop-diffusion. We also found that the diffusion types of IMPs of the outer and inner leaflets were similar with the cases when TMPs were fixed (Fig 7, S12 and S13 Figs). The results are discussed in detail in Supporting Information (S1 Text).

## Discussion

Our results clearly illustrate that the periodic APMS is capable of hindering the diffusion of TMPs and IMPs of the inner leaflet, but the “fence” effect of the AMPS is not sufficient to completely cease diffusion. We also showed that diffusion of lipids and IMPs of the outer leaflet is not directly affected by the APMS. Accumulated TMPs can immobilize the diffusion of all axonal membrane proteins and lipids. This is a poignant result because the periodic structure of the APMS exists in both the AIS and the distal axon, but the diffusion barrier is only observed in the AIS. We propose that accumulation of TMPs in the AIS is the primary mechanism through which axonal membrane proteins and lipids are immobilized. However, the effects of the APMS and accumulation of TMPs on APM protein diffusion are not mutually exclusive and an intact AIS structure is necessary for the existence of the diffusion barrier. Indeed, Song and colleagues experimentally demonstrated that loss of ankyrin G and the resulting disruption of the APMS leads to a vanishing of the diffusion barrier in the AIS [17]. In addition, the diffusion barrier is impaired when actin is disrupted [16, 18].

We propose the following process for the formation of the diffusion barrier in the AIS. First, during early neuronal development, when the periodic actin/spectrin cytoskeleton emerges before the accumulation of ankyrin G, the membrane proteins in the AIS diffuse freely until encountering the corral’s boundaries. These actin and spectrin “fences” restrict the motion of membrane proteins within the corrals but do not immobilize membrane proteins. Second, during the stabilization of periodic cytoskeleton, ankyrin G proteins start accumulating in the AIS, and ankyrin G-anchored proteins (e.g., Na_v_ channels, K_v_ channels, NF 186, and NrCAM) are recruited. Thus, the motion of those membrane proteins are confined or immobilized by anchoring to ankyrin G. Finally, the accumulation of ankyrin G-anchored proteins and other TMPs in the AIS of a mature neuron act as “pickets”, forming a membrane environment that immobilizes all membrane proteins and lipids.

Another important conclusion of this work is that diffusion of axon plasma membrane proteins is deeply anisotropic, as longitudinal diffusion is of different type than transverse diffusion. Our model predicts that longitudinal diffusion of all diffusing axon plasma proteins (IMPs of the inner and outer layer, and TMPs) is confined diffusion because actin rings act as impenetrable “fences”. This is in agreement with results shown in Albrecht et al. [20] where the motion of IMPs of the outer layer is confined within periodic stripes with boundaries overlapping with actin rings. The case of transverse diffusion however is different because spectrin tetramers do not interact with IMPs of the outer layer and form a permeable barrier for IMPs of the inner layer and for TMPs. As a result, the transverse diffusion of IMPs of the outer layer is normal whereas the transverse diffusion of IMPS of the inner layer and TMPs can be described as anomalous or hop diffusion. Thus, we predict that the longitudinal diffusion of the IMPs of the outer and inner layer and of TMPs is confined whereas the transverse diffusion is characterized as normal for IMPs of the outer layer and as anomalous or hop diffusion for IMPs of the inner layer and for TMPs. Our computational model can be possibly applied not only for diffusion of axon plasma membrane proteins but also for diffusion of proteins of the outer hair cell lateral wall where a similar actin-spectrin network constraints their mobility and probably induces an anisotropic diffusion [71].

We also note that diffusion anisotropicity in the case of the axon plasma membrane results directly from the anisotropicity of the structure of the APMS and its tethering to the lipid bilayer. This is different than anisotropic diffusion caused by extension or shearing of the plasma membrane as is the case of band-3 protein diffusion in the RBC plasma membrane described in Auth et al. [24]. In this case, the membrane skeleton, which is formed by triangular equilateral corals is isotropic at equilibrium [25, 28]. However, in the deformed RBC membrane the triangular corrals are extended along the principal direction of the deformations allowing larger diffusion in this direction. In addition, deformation causes changes in membrane thermal oscillations and in the association between spectrin tetramers and lipid bilayer resulting in further changes in band-3 directional mobility [24]. Future studies should explore whether deformation of the axon plasma membrane affects diffusion of membrane proteins as a result of changes in the geometry and biophysical behavior of the APMS and its association to the lipid bilayer.

In conclusion, in mature neurons the APM of the AIS functions as a diffusion barrier that ceases the movement of proteins between the soma and axon and contributes to the maintenance of neuronal polarity. Here, we introduce a CGMD model for the APM that includes representations of the APMS, the phospholipid bilayer, TMPs, and IMPs in both the inner and outer lipid layers to investigate the diffusion of lipids and membrane proteins in the APM. We first showed that at low surface density of TMPs, lipid diffusion is not affected by the APMS or by membrane proteins. This finding parallels experimental results observed in early neuronal development. Next, we observed that actin rings limit the longitudinal diffusion of TMPs and the IMPs of the inner leaflet but not of the IMPs of the outer leaflet. To reconcile the experimental observations with our simulations, we conjectured the existence of actin-anchored proteins that form a fence to restrict the longitudinal diffusion of IMPs of the outer leaflet. We also showed that spectrin filaments can modify transverse diffusion of TMPs and IMPs of the inner leaflet, depending on the strength of the association between lipids and spectrin. In the AIS, where spectrin and lipids could associate due to the presence of pleckstrin domain in spectrin IV, spectrin filaments completely restrict diffusion of proteins within the skeleton corrals and are thus likely to contribute to the immobilization of proteins during the later stage of neural development. In the distal axon, where spectrin and lipids do not associate, spectrin affects diffusion of membrane proteins in a more subtle way via a steric effect. Specifically, spectrin modifies the diffusion of TMPs and IMPs of the inner leaflet from normal to confined-hop diffusion. Finally, we simulated the effect that accumulation of TMPs has on the diffusion of TMPs and IMPs of both the inner and outer leaflets by changing the density of TMPs. We showed that the APMS structure (i.e., actin rings and spectrin filaments) acts as a fence that restricts the diffusion of TMPs and IMPs of the inner leaflet within the membrane skeleton corrals. However, these fences are not sufficient to completely cease diffusion of membrane proteins. Accumulation of TMPs in the AIS is the primary source of membrane protein immobilization. In particular, a ~30-fold increase in TMP density corresponding to only ~25% of surface coverage blocks diffusion of lipids and membrane proteins due to accumulation of TMPs. Overall, we showed that diffusion of axon plasma membrane proteins is anisotropic as longitudinal diffusion is of different type than transverse (azimuthal) diffusion. Our findings provide insight into how protein and lipid diffusion is controlled in the AIS to allow neurons to effectively send and receive electrical signals.

## Supporting information

S1 Text Interfacial tension, bending rigidity of lipid bilayer, and effect of accumulation of mobile TMPs on the diffusion of membrane proteins and lipids in the APM of the AIS. (DOCX)

S1 Fig Potentials used in the simulations. (A) Representation of the sizes of actin, spectrin, and TMP. (B) Translational and rotational coordinates of a particle. (C) Potentials used in the APMS. S represents spectrin, K represents a Na_v_ channel anchored to ankyrin G, A represents actin, and G represents an actin-anchored protein. (D) Membrane potential between two lipid particles with different relative orientations **n**_*i*_. (see main text for more details).
(TIF)

S2 Fig Interfacial tension of the phospholipid bilayer with respect to values of the parameter k employed in Eq 3 that describes the interaction between particles belonging to the two different leaflets. (TIF)

S3 Fig Vertical displacement fluctuation spectrum of membrane model as a function of the dimensionless quantity *qσ*/*π*, where *q* is the wave number and *σ* is the length scale. (TIF)

S4 Fig Unwrapping of the cylindrical coordinates to the corresponding Cartesian coordinates. (A) A cylindrical surface is unwrapped to a flat plane. (B) Point P with cylindrical coordinates with respect to point O are (*r*,*θ*,*z*) on a circle is unwrapped to point P’ on the plane with coordinates (*x* = *rθ*,*y* = 0,*z*).
(TIF)

S5 Fig Trajectories of two IMPs of the outer leaflet projected to a plane in the case where there are no actin-anchored proteins. Note the lack of a stripe formation.
(TIF)

S6 Fig Effect of association to actin on the diffusion of IMPs of the outer leaflet. (A) Computational model in the case of association between actin and IMPs of the outer leaflet, where one IMP particle (green) is restricted by only one actin-associated proteins (yellow). Each association pair is marked as black dash box. (B) Overall MSDs of the outer leaflet IMPs with and without the association.
(TIF)

S7 Fig MSDs along the longitudinal direction as functions of time for (A) IMPs of the inner leaflet and (B) TMPs. Red dashed line fits the data to confined hop diffusion, whereas the black dash line fits the data to anomalous diffusion. Confined diffusion is a better fit for the data.
(TIF)

S8 Fig MSDs of lipids and IMPs of the outer leaflet at longitudinal and transverse directions at various spectrin-lipid associations as functions of time. (A) *D*_*lipid*,*n* = 0_ = 1.14×10^−2^*σ*^2^/*t*_*s*_ *D*_*lipid*,0.02_ = 1.09×10^−2^*σ*^2^/*t*_*s*_ *D*_*lipid*,0.05_ = 1.07×10^−2^*σ*^2^/*t*_*s*_. (B) *D*_*outer*,*n* = 0_ = 3.84×10^−3^*σ*^2^/*t*_*s*_ *D*_*outer*,0.02_ = 3.77×10^−2^*σ*^2^/*t*_*s*_ *D*_*outer*,0.05_ = 3.65×10^−2^*σ*^2^/*t*_*s*_.
(TIF)

S9 Fig MSDs of IMPs of the inner leaflet and TMPs at the transverse direction at n = 0. The black dashed line is the anomalous diffusion fitting with A*t^b^. The red dashed line is the curve fitting of the confined hop diffusion. (A) Squared norm of the residual of the transversal MSD of IMPs in the inner leaflet 2.25×10^7^ (anomalous diffusion) and 5.37×10^6^ (confined diffusion). (B) Squared norm of the residual of the transversal MSD of TMPs 4.59×10^6^ (anomalous diffusion) and 2.87×10^6^ (confined diffusion).
(TIF)

S10 Fig Illustration of the density of TMPs (A) pprc = 3 (0.88% of the surface area) and (B) pprc = 25 (7.28% of the surface area).
(TIF)

S11 Fig **MSDs of lipids (A) and IMPs (B) of the outer layer as functions of time when TMPs are anchored to the APMS.** The blue line represents the case of pprc = 3 (0.88% of the surface area). The green line represents the case of pprc = 20 (5.83% of the surface area). The black line represents the case of pprc = 45 (13.11% of the surface area). The magenta line represents the case of pprc = 60 (17.48% of the surface area). The red line represents the case of pprc = 90 (26.22% of the surface area).
(TIF)

S12 Fig One-dimensional MSDs of TMPs and IMPs of the inner leaflets at different TMP densities. (A) Longitudinal and (B) Transverse MSDs of IMPs of the inner leaflet at 3, 20, 45, 60 and 90 pprc. (C) Longitudinal and (D) Transverse MSDs of TMPs at 3, 20, 45, 60 and 90 pprc.
(TIF)

S13 Fig **MSDs of lipids (A) and IMPs (B) of the outer layer as functions of time when TMPs are not anchored to the APMS.** The blue line represents the case of pprc = 3 (0.88% of the surface area). The green line represents the case of pprc = 20 (5.83% of the surface area). The black line represents the case of pprc = 45 (13.11% of the surface area). The magenta line represents the case of pprc = 60 (17.48% of the surface area). The red line represents the case of pprc = 90 (26.22% of the surface area).
(TIF)

S1 Table Potentials and corresponding parameters used to model the axon plasma membrane. (PDF)

S2 Table Longitudinal diffusion coefficients of membrane proteins and lipids. (PDF)

S3 Table Transverse diffusion coefficients of membrane proteins and lipids. (PDF)

S4 Table Diffusion coefficients of lipids and IMPs of the outer layer for different accumulation densities of immobile TMPs. (PDF)

S5 Table Longitudinal and transverse diffusion coefficients of IMPs of the inner layer for different accumulation densities of immobile TMPs. (PDF)
