# Supplementary material for: Modeling of the axon plasma membrane structure and its effects on protein diffusion
Source: PLoS Comput Biol. 2019 May 2;15(5):e1007003. doi: 10.1371/journal.pcbi.1007003 (PMC6497228; doi:10.1371/journal.pcbi.1007003)
Supplement: S1 Text — (DOCX) [file pcbi.1007003.s001.docx]

**S1 Text**

**Interfacial tension of lipid bilayer**

The stress tensor of a fluid system is invariant in the lateral directions and can be written as

$\sigma_{xy}=\left(\begin{matrix} \sigma_{T} & 0 & 0 \\ 0 & \sigma_{T} & 0 \\ 0 & 0 & \sigma_{N} \end{matrix}\right),$ and the mechanical definition of the interfacial tension is $T_{\mathrm{int}er}=\sigma_{T}-\sigma_{N}$ [1]. The stress tensor from a coarse-grained simulation can be calculated from the expression $\sigma_{xy}=\frac{1}{V}\left[\sum_{j} m_{j}v_{jx}v_{jy}+\frac{1}{2}\sum_{i\neq j} r_{ijx}f_{ijy}\right]$ [2]. We chose the attraction factor k, which controls the attraction between the two lipid layers and is defined in Eqn. 3 in the main text, to be 0.01. For this value of k the interfacial tension between the two lipid layers is approximately zero (see S2 Fig) as it has been suggested by Goetz and Lipowsky [3].

**Bending rigidity of lipid bilayers**

The free energy of a fluid membrane within the framework of continuum mechanics can be written as $F=\int\left[\gamma+\frac{\kappa}{2}(C_{1}+C_{2}-C_{0})^{2}+\bar{\kappa}C_{1}C_{2}\right] dA,$ where $C_{0}$is the spontaneous curvature, $C_{1}$and$C_{2}$are the principal curvature, $\gamma$is the surface tension, $\kappa$is the bending rigidity, and $\bar{\kappa}$is the Gaussian rigidity, according to the Canham-Helfrich theory [4]. Appling the Monge representation and equipartition theorem on the Helfrich free energy, the power spectrum can be written as $\left\langle\left|\tilde{h}\left(\mathbf{q}\right)\right|^{2}\right\rangle=\frac{K_{B}T}{l^{2}\left(\gamma\left|\mathbf{q}\right|^{2}+\kappa\left|\mathbf{q}\right|^{4}\right)},$ whereis the discrete Fourier transform of the out-of-plane displacement$h\left(\mathbf{r}\right)$, $\mathbf{q}=\left(2\pi n_{x}/L,2\pi n_{y}/L\right)$, $q=\left|\mathbf{q}\right|$, $l$ is the size of the coarse-grained lipid particle, and $L$is the size of the simulation box [5]. The proposed membrane model provides a nearly 0 surface tension, which leads to a $q^{-4}$dependence on the wave vector.

By fitting the curve of $\tilde{h}\left(\mathbf{q}\right)$ against $q^{-4}$as shown in S3 Fig, the bending rigidity of the lipid bilayer is calculated to be $12.26K_{B}T$, which lies within the range of measured experimental data for a lipid bilayer $10\sim20K_{B}T$[5, 6].

**Effect of accumulation of mobile TMPs on the diffusion of membrane proteins and lipids in the APM of the AIS**

To study the effect of the accumulation of TMPs, we created different environments by increasing the initial surface density of TMPs from three particles per rectangular corral (ρ=3 pprc) to 20, 45, 60, and 90 pprc. These surface densities of TMPs corresponds to surface area coverages of 0.87%, 5.83%, 13.11%, 17.48%, and 26.22%, respectively.

We measured the MSDs of IMPs of the inner leaflet at the axon’s longitudinal and transverse directions at different TMP densities, with no attraction between spectrin filaments and lipid particles (n=0 in Eqn. 4). As discussed previously, IMPs of the inner leaflet underwent confined longitudinal diffusion. This is apparent in Figure A in S12 Fig and Figure B in S12 Fig for ρ=3 pprc and ρ=20 pprc, where MSDs almost reached their maximum value for the observed number of time steps. Similarly, for ρ=45 pprc, ρ=60 pprc, and ρ=90 pprc, although the longitudinal diffusion was eventually confined, the required number of time steps for the MSDs to reach their maximum value went beyond the observed time period. For example, if we used the characteristic lengths $L=116.4 nm$ and $L=122.1 nm$, computed in the case of ρ=3 pprc for TMPs and IMPs of the inner leaflet, respectively, we found that when ρ=45 pprc, approximately $5\times10^{7}$more time steps were required to reach the maximum MSD value. This meant that at 45 pprc (13.11% area coverage), the motion of TMPs and IMPs of the inner leaflet was significantly hindered and the number of time steps in our simulation was not large enough to see the effect of corrals.

The effect of the accumulation of TMPs was clearly reflected on the value of the microscopic diffusion coefficient, which corresponds to the slope of the linear portion of the MSD graph at small time scale. By fitting the corresponding MSDs with the expression of confined-hop diffusion, we found that as the pprc increased from 3 to 20, 45, 60 and 90, the microscopic diffusion coefficient of both the IMPs of the inner leaflet and TMPs decreased from $D_{inner,3pprc}^{Longitudinal}=3.31\times10^{-3}\sigma^{2}/t_{s}$ and $D_{TMP,3pprc}^{Longitudinal}=1.53\times10^{-3}\sigma^{2}/t_{s}$ to$D_{inner,20pprc}^{Longitudinal}=2.21\times10^{-3}\sigma^{2}/t_{s}$ and $D_{TMP,20pprc}^{Longitudinal}=1.02\times10^{-3}\sigma^{2}/t_{s}$, to $D_{inner,45pprc}^{Longitudinal}=1.88\times10^{-4}\sigma^{2}/t_{s}$ and$D_{TMP,45pprc}^{Longitudinal}=9.75\times10^{-5}\sigma^{2}/t_{s}$, to $D_{inner,60pprc}^{Longitudinal}=1.07\times10^{-4}\sigma^{2}/t_{s}$and $D_{TMP,60pprc}^{Longitudinal}=6.12\times10^{-5}\sigma^{2}/t_{s}$to $D_{inner,90pprc}^{Longitudinal}=2.76\times10^{-5}\sigma^{2}/t_{s}$ and $D_{TMP,90pprc}^{Longitudinal}=1.28\times10^{-5}\sigma^{2}/t_{s}$, respectively.

In transverse diffusion, accumulation of TMPs once again failed to change the nature of diffusion and merely reduced the micro-diffusion coefficient. As discussed above, transverse diffusion can be described as confined hop diffusion. For the values ρ=3, 20, 45, 60 and 90 pprc the micro-diffusion coefficients for the IMPs of the inner leaflet and TMPs decreased from $D_{inner,3pprc}^{Transverse}=3.38\times10^{-3}\sigma^{2}/t_{s}$and$D_{TMP,3pprc}^{Transverse}=1.26\times10^{-3}\sigma^{2}/t_{s}$, to $D_{inner,20pprc}^{Transverse}=2.12\times10^{-3}\sigma^{2}/t_{s}$ and$D_{TMP,20pprc}^{Transverse}=9.54\times10^{-4}\sigma^{2}/t_{s}$, to $D_{inner,45pprc}^{Transverse}=2.04\times10^{-4}\sigma^{2}/t_{s}$and$D_{TMPs,45pprc}^{Transverse}=7.24\times10^{-5}\sigma^{2}/t_{s}$, to $D_{inner,60pprc}^{Transverse}=1.04\times10^{-4}\sigma^{2}/t_{s}$and$D_{TMP,60pprc}^{Transverse}=5.06\times10^{-5}\sigma^{2}/t_{s}$ to$D_{inner,90pprc}^{Transverse}=2.71\times10^{-5}\sigma^{2}/t_{s}$and $D_{TMP,90pprc}^{Transverse}=1.15\times10^{-5}\sigma^{2}/t_{s}$ (Figure B in S12 Fig and Figure D in S12 Fig). The values of the microscopic diffusion coefficient at ρ=90 pprc for both IMPs of the inner leaflet and TMPs correspond to practical immobility of the diffusing particles. This result suggests that accumulated TMPs act as “pickets” that can slow down all lipid and membrane protein diffusion [7, 8]. Finally, in the case of lipids and IMPs of the outer leaflet, the accumulation of TMPs effect simply decreased the diffusion coefficient without modifying the diffusion type (S13 Fig). At ρ=90 pprc, diffusion practically ceased.

**References**

1. Rowlinson JS, Widom B. Molecular theory of capillarity. Oxford: Clarendon Press; 1982. xi, 327 p. p.

2. McQuarrie DA. Statistical mechanics. New York ; London: Harper and Row; 1976. xvii, 641 p. p.

3. Goetz R, Lipowsky R. Computer simulations of bilayer membranes: Self-assembly and interfacial tension. The Journal of Chemical Physics. 1998;108(17):7397-409. doi: 10.1063/1.476160.

4. Canham PB. The minimum energy of bending as a possible explanation of the biconcave shape of the human red blood cell. J Theor Biol. 1970;26(1):61-81. Epub 1970/01/01. PubMed PMID: 5411112.

5. Boal DH. Mechanics of the cell. Cambridge, UK ; New York: Cambridge University Press; 2002. xiv, 406 p. p.

6. Lipowsky R. The morphology of lipid membranes. Curr Opin Struct Biol. 1995;5(4):531-40. Epub 1995/08/01. doi: 0959-440X(95)80040-9 [pii]. PubMed PMID: 8528770.

7. Fujiwara T, Ritchie K, Murakoshi H, Jacobson K, Kusumi A. Phospholipids undergo hop diffusion in compartmentalized cell membrane. J Cell Biol. 2002;157(6):1071-81. Epub 2002/06/12. doi: 10.1083/jcb.200202050. PubMed PMID: 12058021; PubMed Central PMCID: PMC2174039.

8. Kusumi A, Nakada C, Ritchie K, Murase K, Suzuki K, Murakoshi H, et al. Paradigm shift of the plasma membrane concept from the two-dimensional continuum fluid to the partitioned fluid: high-speed single-molecule tracking of membrane molecules. Annu Rev Biophys Biomol Struct. 2005;34:351-78. Epub 2005/05/05. doi: 10.1146/annurev.biophys.34.040204.144637. PubMed PMID: 15869394.
