# Supplementary figures and images for: Modeling of the axon plasma membrane structure and its effects on protein diffusion
Source: PLoS Comput Biol. 2019 May 2;15(5):e1007003. doi: 10.1371/journal.pcbi.1007003 (PMC6497228; doi:10.1371/journal.pcbi.1007003)

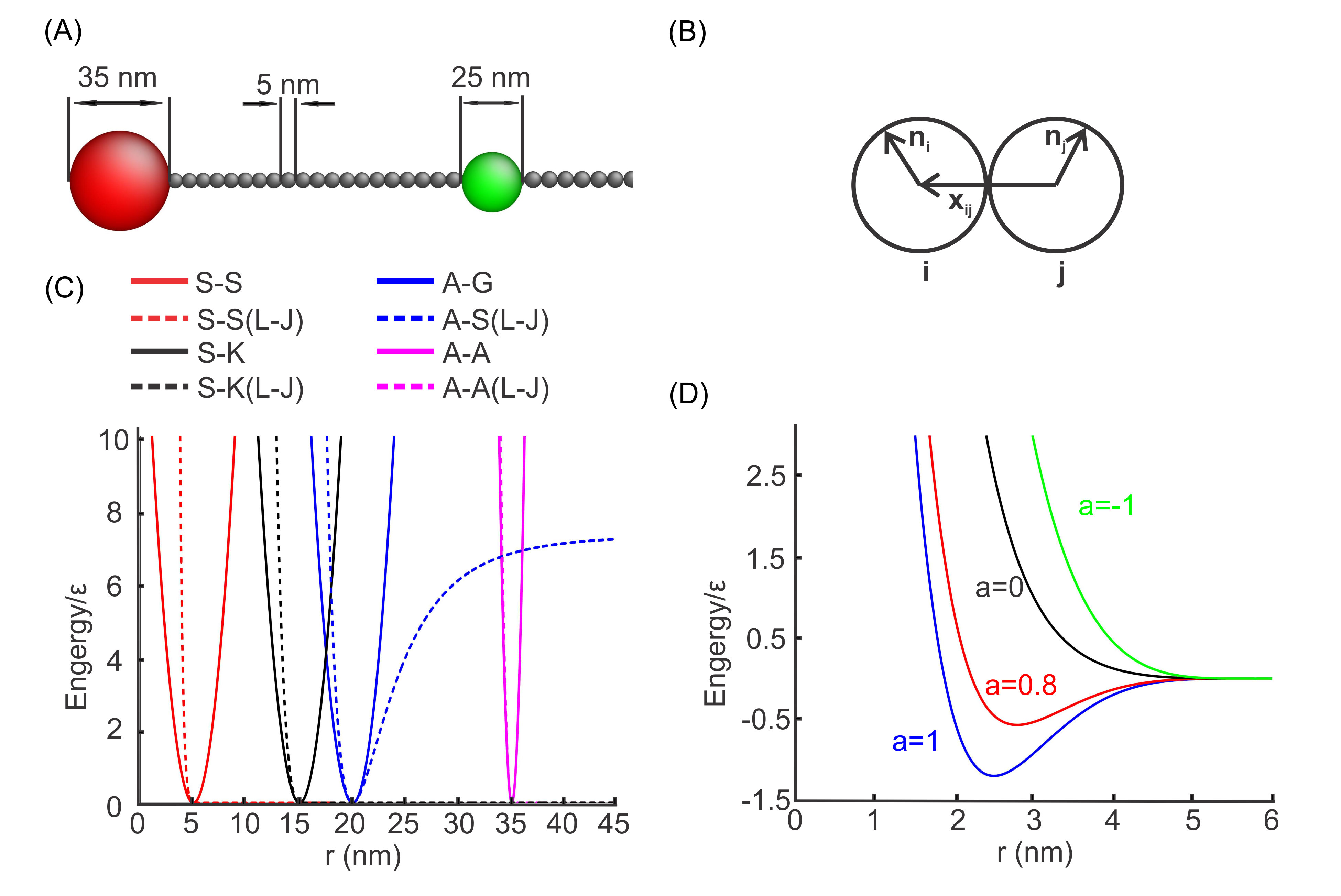

Supplement: S1 Fig — (A) Representation of the sizes of actin, spectrin, and TMP. (B) Translational and rotational coordinates of a particle. (C) Potentials used in the APMS. S represents spectrin, K represents a Nav channel anchored to ankyrin G, A represents actin, and G represents an actin-anchored protein. (D) Membrane potential between two lipid particles with different relative orientations ni. (see main text for more details). (TIF) [file pcbi.1007003.s002.tif]

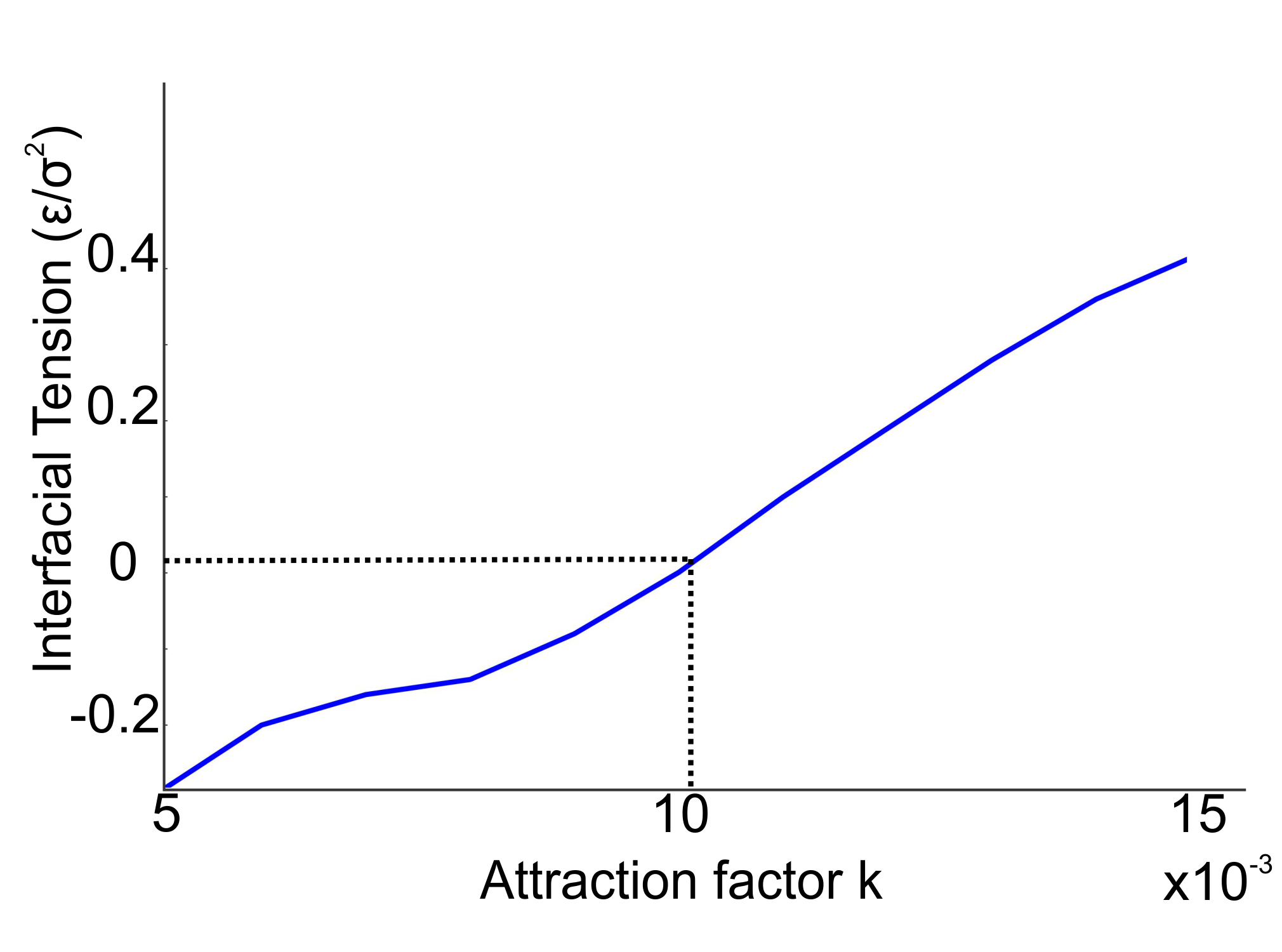

Supplement: S2 Fig — (TIF) [file pcbi.1007003.s003.tif]

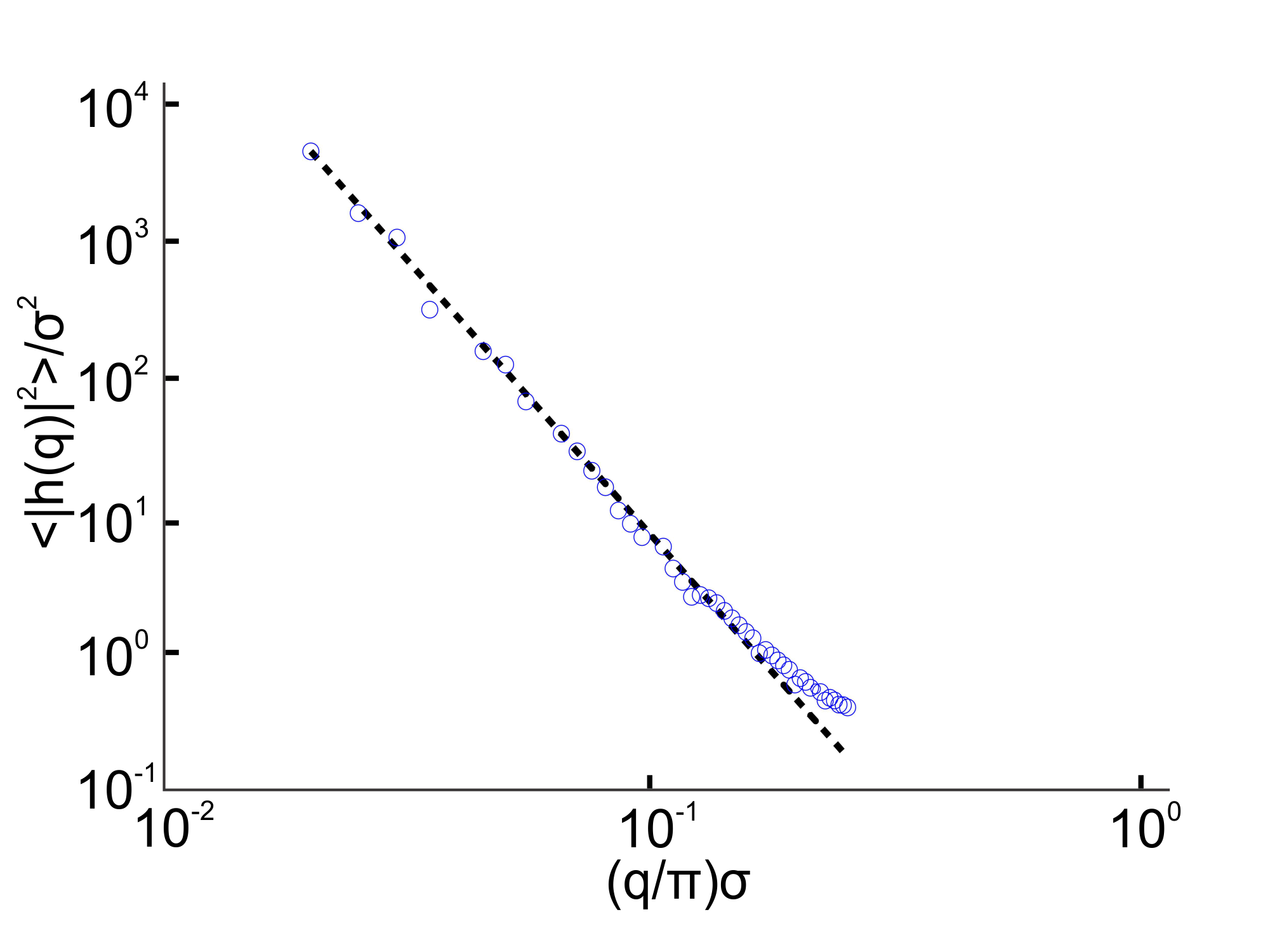

Supplement: S3 Fig — (TIF) [file pcbi.1007003.s004.tif]

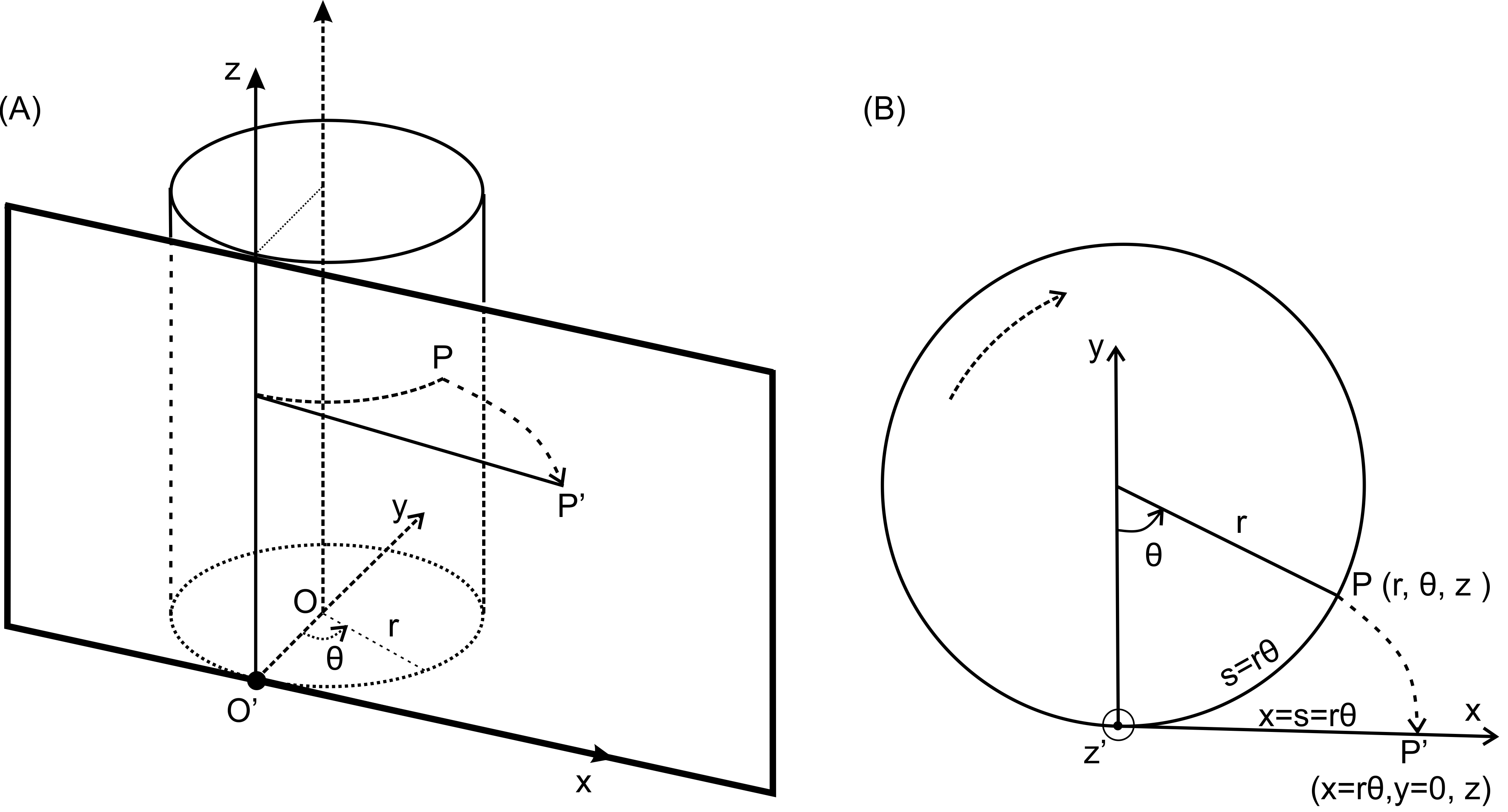

Supplement: S4 Fig — (A) A cylindrical surface is unwrapped to a flat plane. (B) Point P with cylindrical coordinates with respect to point O are (r,θ,z) on a circle is unwrapped to point P’ on the plane with coordinates (x = rθ,y = 0,z). (TIF) [file pcbi.1007003.s005.tif]

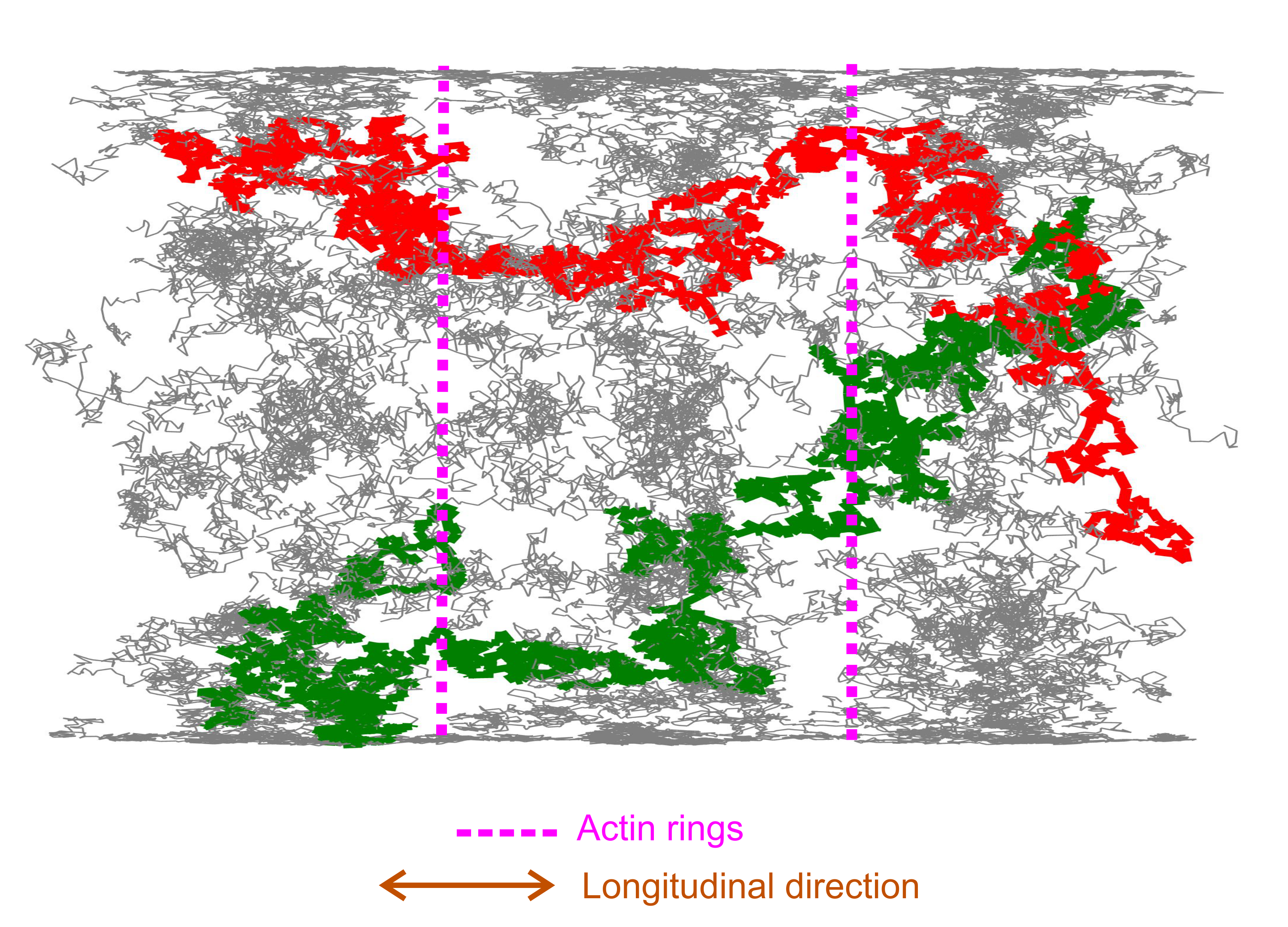

Supplement: S5 Fig — Note the lack of a stripe formation. (TIF) [file pcbi.1007003.s006.tif]

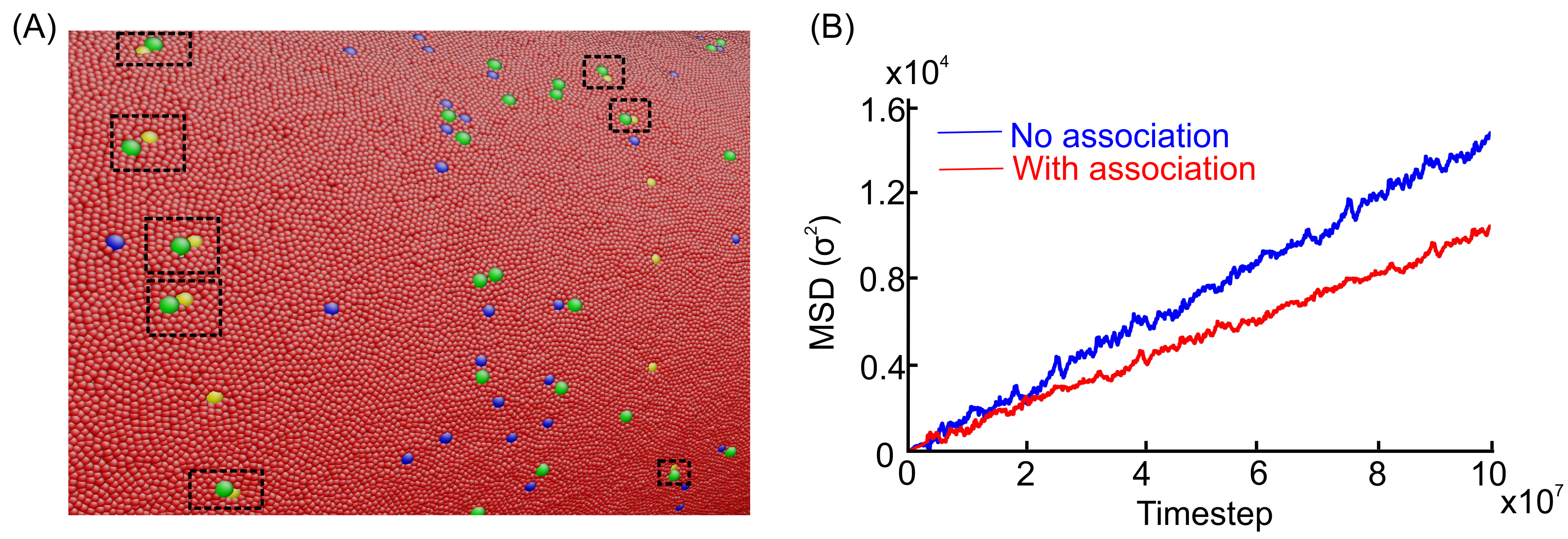

Supplement: S6 Fig — (A) Computational model in the case of association between actin and IMPs of the outer leaflet, where one IMP particle (green) is restricted by only one actin-associated proteins (yellow). Each association pair is marked as black dash box. (B) Overall MSDs of the outer leaflet IMPs with and without the association. (TIF) [file pcbi.1007003.s007.tif]

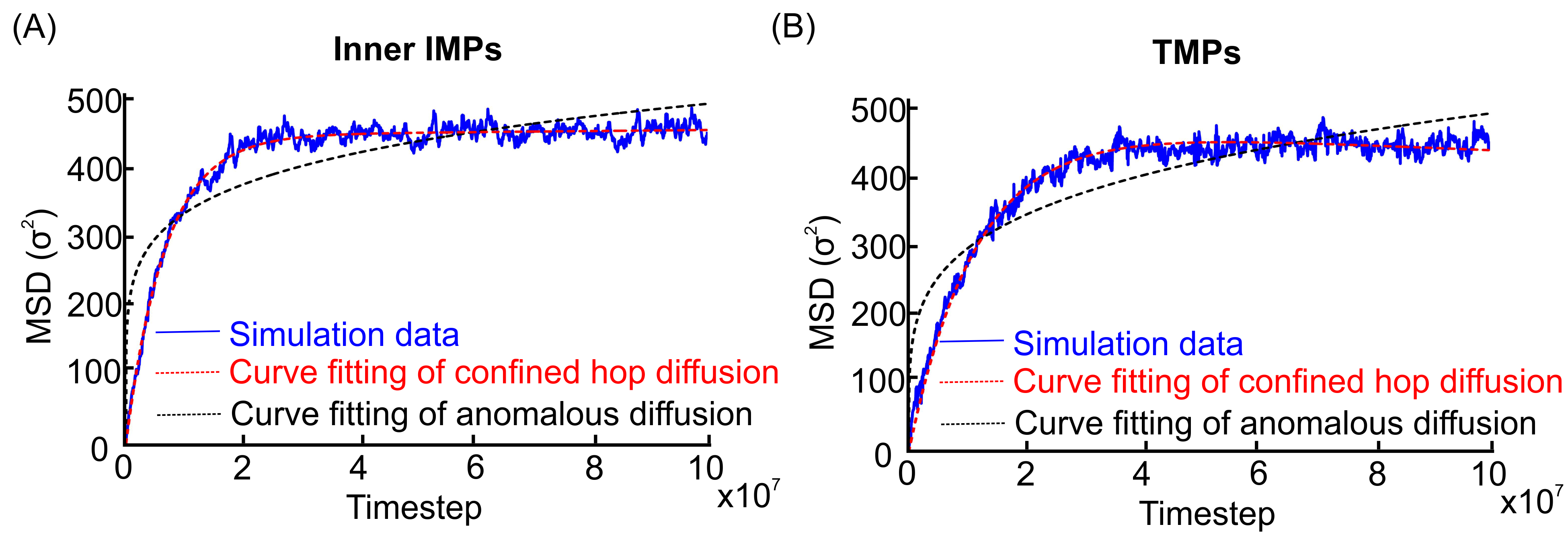

Supplement: S7 Fig — MSDs along the longitudinal direction as functions of time for (A) IMPs of the inner leaflet and (B) TMPs. Red dashed line fits the data to confined hop diffusion, whereas the black dash line fits the data to anomalous diffusion. Confined diffusion is a better fit for the data. (TIF) [file pcbi.1007003.s008.tif]

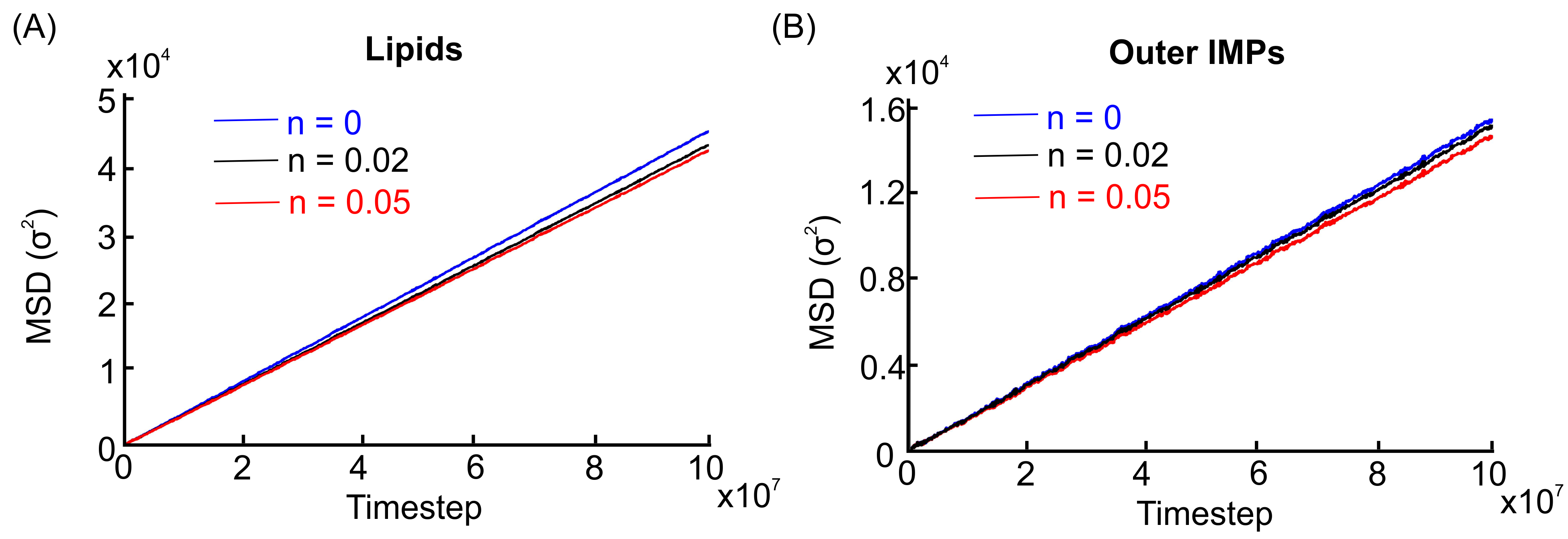

Supplement: S8 Fig — (A) Dlipid,n = 0 = 1.14×10^−2^σ2/ts Dlipid,0.02 = 1.09×10^−2^σ2/ts Dlipid,0.05 = 1.07×10^−2^σ2/ts. (B) Douter,n = 0 = 3.84×10^−3^σ2/ts Douter,0.02 = 3.77×10^−2^σ2/ts Douter,0.05 = 3.65×10^−2^σ2/ts. (TIF) [file pcbi.1007003.s009.tif]

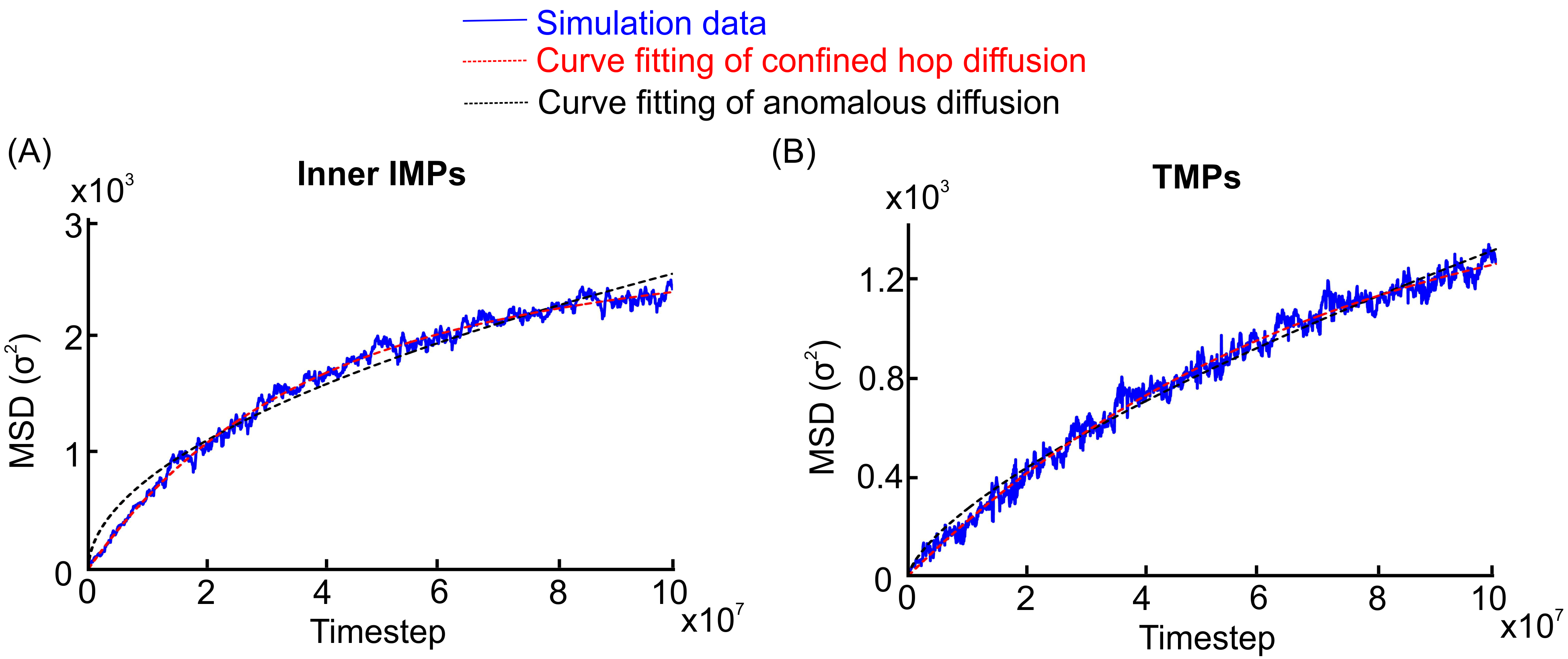

Supplement: S9 Fig — The black dashed line is the anomalous diffusion fitting with A*tb. The red dashed line is the curve fitting of the confined hop diffusion. (A) Squared norm of the residual of the transversal MSD of IMPs in the inner leaflet 2.25×10^7^ (anomalous diffusion) and 5.37×10^6^ (confined diffusion). (B) Squared norm of the residual of the transversal MSD of TMPs 4.59×10^6^ (anomalous diffusion) and 2.87×10^6^ (confined diffusion). (TIF) [file pcbi.1007003.s010.tif]

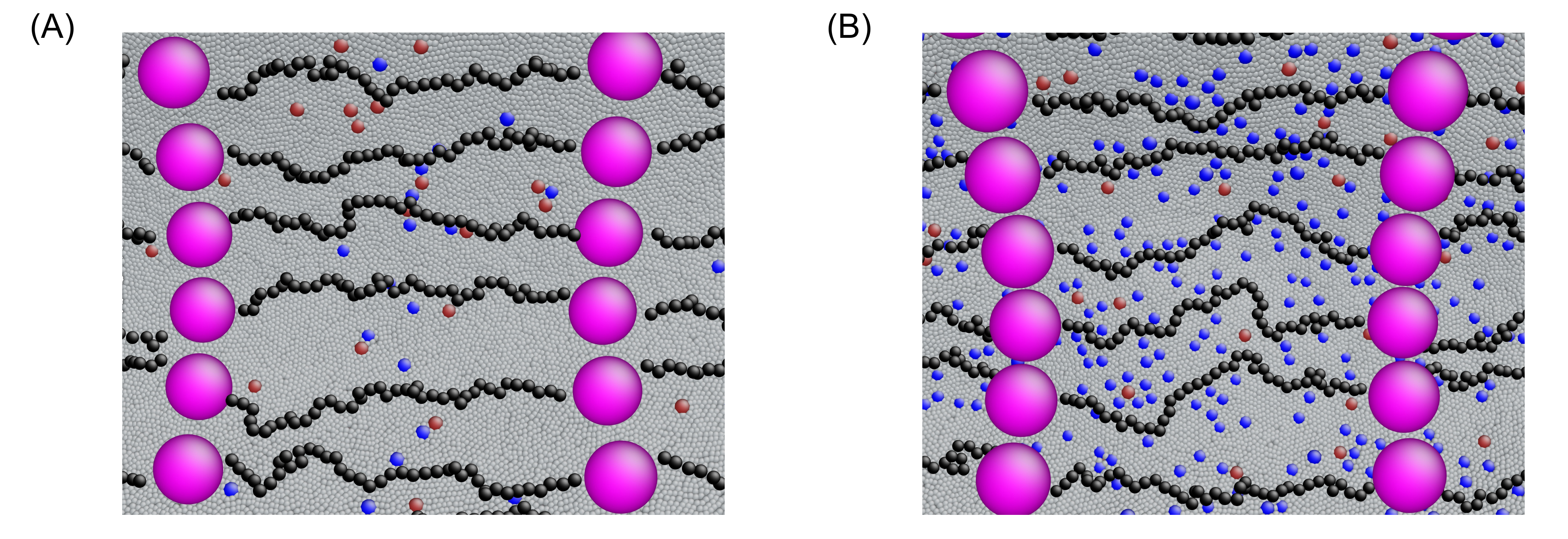

Supplement: S10 Fig — Illustration of the density of TMPs (A) pprc = 3 (0.88% of the surface area) and (B) pprc = 25 (7.28% of the surface area). (TIF) [file pcbi.1007003.s011.tif]

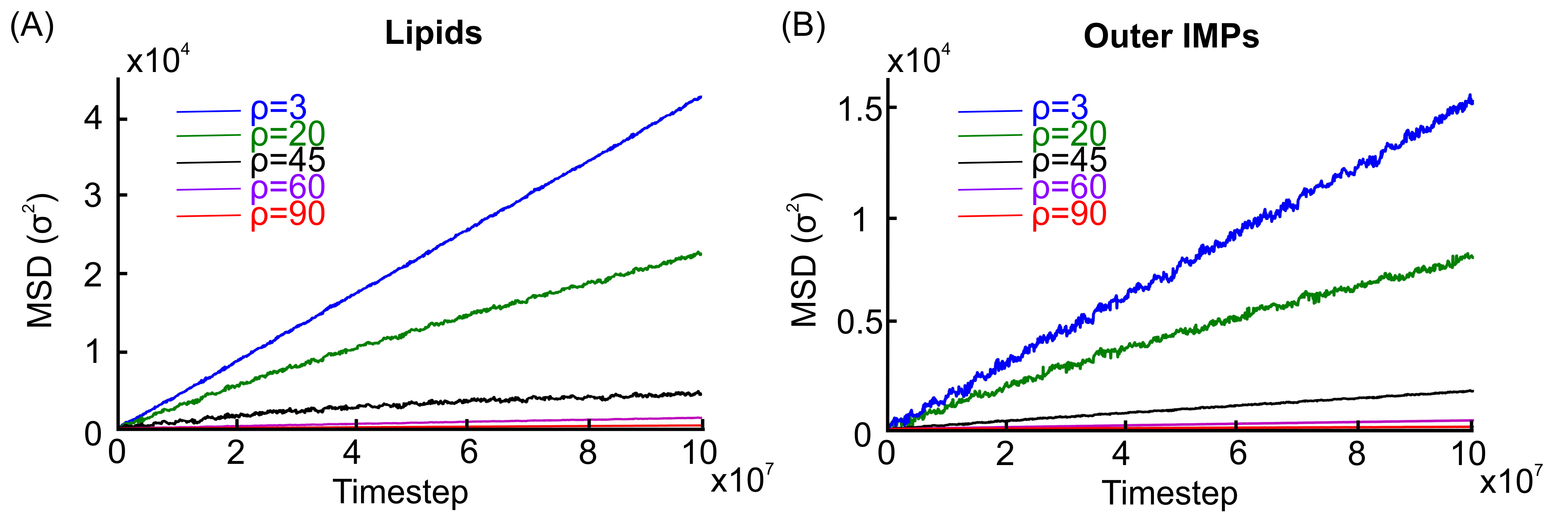

Supplement: S11 Fig — MSDs of lipids (A) and IMPs (B) of the outer layer as functions of time when TMPs are anchored to the APMS. The blue line represents the case of pprc = 3 (0.88% of the surface area). The green line represents the case of pprc = 20 (5.83% of the surface area). The black line represents the case of pprc = 45 (13.11% of the surface area). The magenta line represents the case of pprc = 60 (17.48% of the surface area). The red line represents the case of pprc = 90 (26.22% of the surface area). (TIF) [file pcbi.1007003.s012.tif]

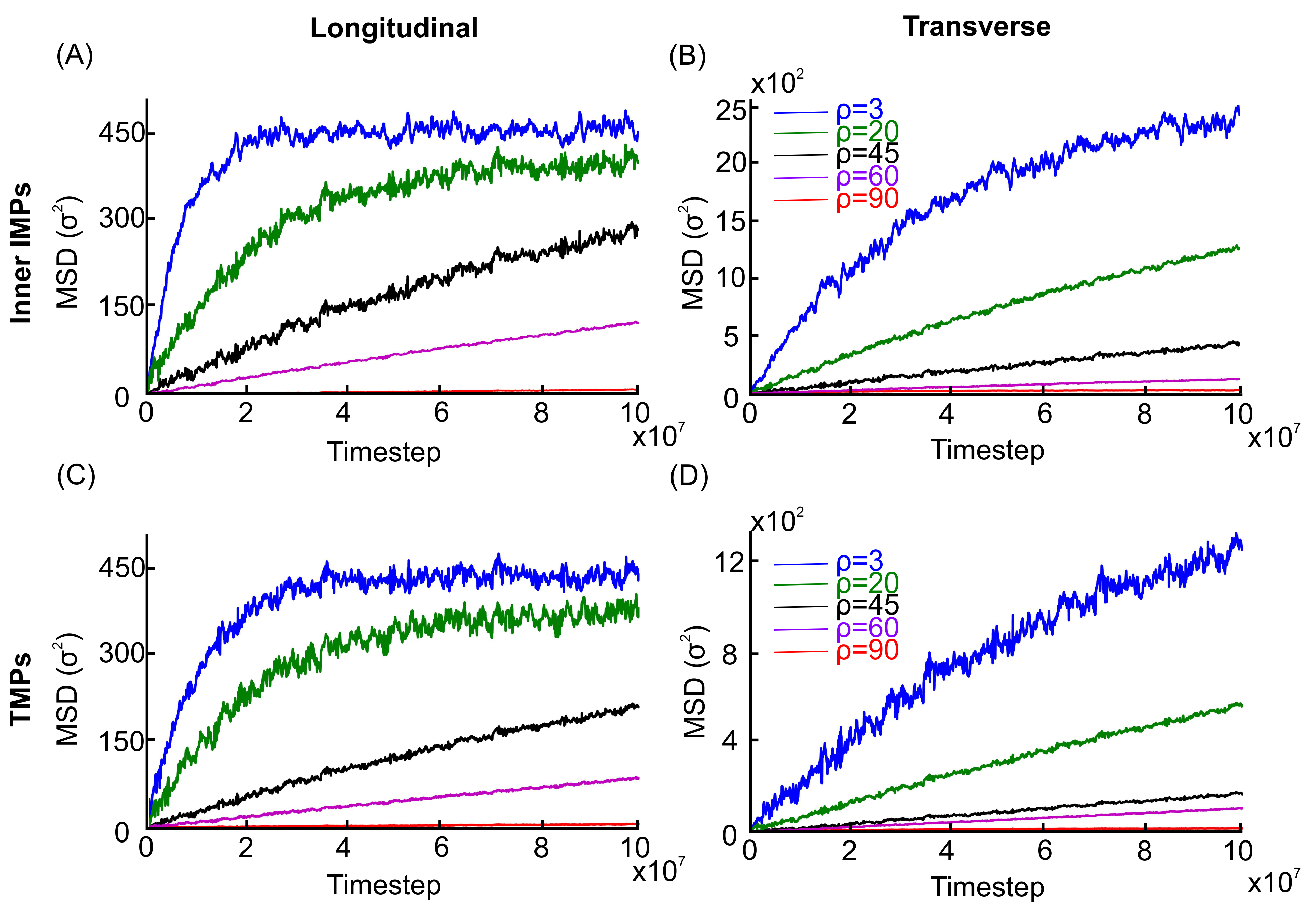

Supplement: S12 Fig — (A) Longitudinal and (B) Transverse MSDs of IMPs of the inner leaflet at 3, 20, 45, 60 and 90 pprc. (C) Longitudinal and (D) Transverse MSDs of TMPs at 3, 20, 45, 60 and 90 pprc. (TIF) [file pcbi.1007003.s013.tif]

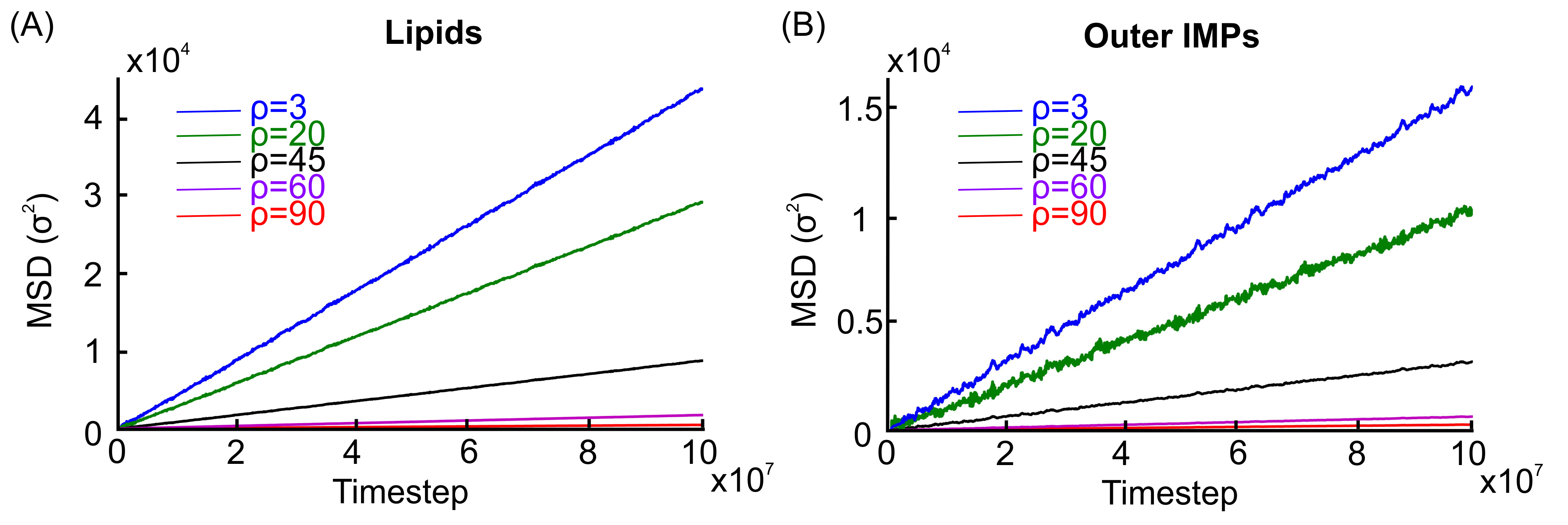

Supplement: S13 Fig — MSDs of lipids (A) and IMPs (B) of the outer layer as functions of time when TMPs are not anchored to the APMS. The blue line represents the case of pprc = 3 (0.88% of the surface area). The green line represents the case of pprc = 20 (5.83% of the surface area). The black line represents the case of pprc = 45 (13.11% of the surface area). The magenta line represents the case of pprc = 60 (17.48% of the surface area). The red line represents the case of pprc = 90 (26.22% of the surface area). (TIF) [file pcbi.1007003.s014.tif]
