# Supplementary material for: Modeling of the axon plasma membrane structure and its effects on protein diffusion
Source: PLoS Comput Biol. 2019 May 2;15(5):e1007003. doi: 10.1371/journal.pcbi.1007003 (PMC6497228; doi:10.1371/journal.pcbi.1007003)
Supplement: S1 Table — (PDF) [file pcbi.1007003.s015.pdf]

S1 Table. Potentials and corresponding parameters used to model the axon plasma membrane.

**Potentials**	
Spring potential	$U_{Spring}(r)=1/2\,K(r-r_0)^2$
Lennard-Jones potential	$U_{LJ}(r)=4\mathrm{E}\left[(S/r)^{12}-(S/r)^{6}\right]+\mathrm{E}$
Bending FENE potential	$U_{bending}=-\frac{1}{2}k_b\,\Delta\theta_{max}\,ln\left[1-\left(\frac{\theta-\theta_0}{\Delta\theta_{max}}\right)^2\right]$
FENE potential between actin rings	$U_{mt}=-\frac{1}{2}k_{mt}\,\Delta d_{max}\,ln\left[1-\left(\frac{d-d_{eq}^{RR}}{\Delta d_{max}}\right)^2\right]$
Membrane potential	$U_{mem}(r_{\mathrm{ij}})=\begin{cases}u_R(r_{ij})=K_mE_m\left((R_{cut,mem}-r_{ij})/(R_{cut,mem}-r_{eq})\right)\\u_A(r_{ij})=-2K_mE_m\left((R_{cut,mem}-r_{ij})/(R_{cut,mem}-r_{eq})\right)^4\end{cases}$

**Parameters used in potentials**			
**Actin-Actin**		**Actin-Spectrin**	
$U^{AA}(r)$	$K=69.35\,\varepsilon/\sigma^2$	$U_{LJ}^{AS}(r)$	$E=7.3\varepsilon$
	$r_0=2^{1/6}\times 14\sigma$		$S=8\sigma$
$U_{LJ\,(rep)}^{AA}(r)$	$E=238.47\,\varepsilon$	**Actin ring-Actin ring**	
	$S=14\sigma$		
$U_{bending}$	$k_b=3500\,K_BT$	U_{mt}	$k_{mt}=19{,}822\,K_BT/d_{eq}^{RR}$
	$\theta_0\simeq 170.77^\circ$		$d_{eq}^{RR}=185\,nm$
	$\Delta\theta_{max}=0.3\,\theta_0$		$\Delta d_{\max}=0.3\,d_{eq}^{RR}$
**Spectrin-Spectrin**		**Spectrin-Ankyrin**	
$U^{SS}(r)$	$K=6.5\,\varepsilon/\sigma^2$	$U^{SK}(r)$	$K=6.5\,\varepsilon/\sigma^2$
	$r_0=2^{1/6}\times 2\sigma$		$r_0=2^{1/6}\times 6\sigma$

$U_{LJ(rep)}^{SS}(r)$	$E = 0.46\varepsilon$	$U_{LJ(rep)}^{SK}(r)$	$E = 4.1\varepsilon$
	$S = 2\sigma$		$S = 6\sigma$
**Actin-Actin-anchored proteins**		**Actin-Lipids**	
$U^{AG}(r)$	$K = 6.5\,\varepsilon/\sigma^2$	$U_{LJ(rep)}^{AL}(r)$	$E = 6.41\varepsilon$
	$r_0 = 2^{1/6} \times 8\sigma$		$S = 7.5\sigma$
**Spectrin-Transmembrane proteins**		**Spectrin-Integral monotopic proteins**	
$U_{LJ(rep)}^{ST}(r)$	$E = 4.1\varepsilon$	$U_{LJ(rep)}^{SI}(r)$	$E = 2.85\varepsilon$
	$S = 6\sigma$		$S = 5\sigma$
**Lipid-Lipid**		**Lipid-Axonal membrane proteins**	
$U_{mem}^{LL}(r)$	$K_m = 1.2$	$U_{mem}^{LP}(r)$	$K_m = 2.8$
	$E_m = \varepsilon$		$E_m = \varepsilon$
	$R_{cut,mem} = 2.6\sigma$		$R_{cut,mem} = 2.6\sigma$
	$r_{eq} = 2^{1/6}\sigma$		$r_{eq} = 2^{1/6} \times 1.5\sigma$
