# Supplementary material for: Modeling of the axon plasma membrane structure and its effects on protein diffusion
Source: PLoS Comput Biol. 2019 May 2;15(5):e1007003. doi: 10.1371/journal.pcbi.1007003 (PMC6497228; doi:10.1371/journal.pcbi.1007003)
Supplement: S2 Table — (PDF) [file pcbi.1007003.s016.pdf]

S2 Table. Longitudinal diffusion coefficients of membrane proteins and lipids.

Diffusion coefficients (σ^2 / t_s)	TMPs	IMPs of the inner leaflet	Lipids	IMPs of the outer leaflet
D_{micro}	1.53×10^{-3}	3.31×10^{-3}	1.13×10^{-2}	3.86×10^{-3}
D_{macro}	0	0		
