# Supplementary material for: Modeling of the axon plasma membrane structure and its effects on protein diffusion
Source: PLoS Comput Biol. 2019 May 2;15(5):e1007003. doi: 10.1371/journal.pcbi.1007003 (PMC6497228; doi:10.1371/journal.pcbi.1007003)
Supplement: S3 Table — (PDF) [file pcbi.1007003.s017.pdf]

S3 Table. Transverse diffusion coefficients of membrane proteins and lipids.

Diffusion coefficients (σ^2 / t_s)	TMPs	IMPs of the inner leaflet	Lipids	IMPs of the outer leaflet
n=0				
D_{micro}	1.26×10^{-3}	3.38×10^{-3}	1.15×10^{-2}	3.82×10^{-3}
D_{macro}	8.97×10^{-5}	1.86×10^{-4}		
n=0.02				
D_{micro}	1.34×10^{-3}	2.94×10^{-3}	1.09×10^{-2}	3.77×10^{-2}
D_{macro}	1.62×10^{-5}	2.54×10^{-5}		
n=0.05				
D_{micro}	1.44×10^{-3}	3.04×10^{-3}	1.07×10^{-2}	3.65×10^{-2}
D_{macro}	2.31×10^{-5}	2.31×10^{-5}		
