# Supplementary material for: Modeling of the axon plasma membrane structure and its effects on protein diffusion
Source: PLoS Comput Biol. 2019 May 2;15(5):e1007003. doi: 10.1371/journal.pcbi.1007003 (PMC6497228; doi:10.1371/journal.pcbi.1007003)
Supplement: S4 Table — (PDF) [file pcbi.1007003.s018.pdf]

S4 Table. Diffusion coefficients of lipids and IMPs of the outer layer for different accumulation densities of immobile TMPs.

Particles per rectangular corral (pprc) (ρ)	Diffusion coefficients of lipids (σ^2 / t_s)	Diffusion coefficients of IMPs of the outer leaflet (σ^2 / t_s)
3	1.14×10^{-2}	3.84×10^{-3}
20	6.08×10^{-3}	2.36×10^{-3}
45	2.01×10^{-3}	7.43×10^{-4}
60	4.28×10^{-4}	1.41×10^{-4}
90	3.79×10^{-5}	2.24×10^{-5}
