# Supplementary material for: Modeling of the axon plasma membrane structure and its effects on protein diffusion
Source: PLoS Comput Biol. 2019 May 2;15(5):e1007003. doi: 10.1371/journal.pcbi.1007003 (PMC6497228; doi:10.1371/journal.pcbi.1007003)
Supplement: S5 Table — (PDF) [file pcbi.1007003.s019.pdf]

S5 Table. Longitudinal and transverse diffusion coefficients of IMPs of the inner layer for different accumulation densities of immobile TMPs.

Particles per rectangular corral (pprc) (ρ)	Longitudinal diffusion coefficients (σ^2 / t_s)	Transverse diffusion coefficients (σ^2 / t_s)
3	3.26×10^{-3}	3.28×10^{-3}
20	2.04×10^{-3}	2.13×10^{-3}
45	1.65×10^{-4}	1.43×10^{-4}
60	9.87×10^{-5}	8.76×10^{-5}
90	2.63×10^{-5}	2.66×10^{-5}
